# Diet and diet-related challenges with specific focus on carbohydrates and carbohydrate counting in adults with type 1 diabetes: a cross-sectional study

**DOI:** 10.1136/bmjopen-2025-101619

**Published:** 2025-11-29

**Authors:** Sofia Sterner Isaksson, Jarl Hellman, Magnus Wijkman, Henrik Imberg, Arndís Finna Ólafsdóttir, Mette Axelsen, Marcus Lind

**Affiliations:** 1 Department of Molecular and Clinical Medicine, Institute of Medicine, Sahlgrenska Academy, University of Gothenburg, Gothenburg, Västra Götaland, Sweden; 2 Department of Medicine, NU Hospital Group, Uddevalla, Sweden; 3 Department of Medical Sciences, Uppsala University, Uppsala, Sweden; 4 Department of Internal Medicine and Department of Health, Medicine and Caring Sciences, Linköping University, Norrköping, Sweden; 5 Statistiska Konsultgruppen Sweden, Gothenburg, Sweden; 6 Institute of Medicine, Sahlgrenska Academy, University of Gothenburg, Gothenburg, Västra Götaland, Sweden; 7 Global Oatly Science and Innovation Centre, Lund, Sweden; 8 Department of Medicine, Geriatrics and Emergency Care, Sahlgrenska University Hospital, Gothenburg, Västra Götaland, Sweden

**Keywords:** Surveys and Questionnaires, DIABETES & ENDOCRINOLOGY, NUTRITION & DIETETICS, Observational Study

## Abstract

**Abstract:**

**Objectives:**

To explore dietary intake, diet-related challenges in glucose management and perceived needs for dietary support among Swedish adults with type 1 diabetes (T1D).

**Design:**

Cross-sectional observational study based on an electronic survey that included the validated Meal-Q food frequency questionnaire and additional questions on dietary habits and management. Participant characteristics were retrieved from the Swedish National Diabetes Register. Descriptive and correlation analyses were conducted.

**Setting:**

Three diabetes specialist clinics in Sweden.

**Participants:**

375 adults with T1D.

**Main outcome measures:**

Dietary intake and diet-related challenges in glucose management.

**Results:**

A total of 191 persons (mean age 48 years; 48% female) consented to participate. The mean (SD) glycated haemoglobin A1c was 56 (13) mmol/mol, mean glucose 8.8 (2.2) mmol/L, time in range (TIR) 64% (18%) and BMI 27 (4.3) kg/m²; 41% used insulin pumps. Mean carbohydrate intake was 183 g/day (41% of energy, E%). Fibre intake was 23 g/day (3.1 g/MJ), and saturated fat intake was 29 g/day (15 E%), both inconsistent with dietary recommendations. About half (51%) found carbohydrate counting challenging, with 53% estimating carbohydrate intake visually and only 18% using advanced methods. Additionally, 48% reported reducing carbohydrate intake, and 61% avoided certain carbohydrate-rich foods due to glucose management difficulties. Approximately 40% of participants reported insufficient dietary guidance from their healthcare providers since diagnosis, 33% expressed interest in further dietitian support and 39% believed dietary changes could improve glucose control.

**Conclusions:**

Participants reported lower fibre intake and higher saturated fat intake compared with dietary guidelines. Many found carbohydrate counting and carbohydrate-rich meals challenging. One-third expressed a wish for additional dietary support. These findings highlight the importance of improving access to tailored dietary counselling in routine T1D care.

**STRENGTHS AND LIMITATIONS OF THIS STUDY**

- Participants were randomly selected from three diabetes specialist centres, enhancing representativeness and reducing selection bias.
- The use of a validated food frequency questionnaire (Meal-Q) strengthened dietary assessment reliability.
- Self-reported dietary intake is subject to recall bias and potential under-reporting, particularly regarding energy intake.
- The cross-sectional design allows only observational interpretation, and the moderate response rate (51%) may affect generalisability to the broader type 1 diabetes population.

## Introduction

 Effective glucose control significantly reduces complications and lowers mortality in individuals with type 1 diabetes (T1D).^1 2^ Despite advances in care, mortality rates remain higher in this population compared with the general population,^3^,^5^ underscoring the need for substantial improvement in managing cardiovascular risk factors, including hyperglycaemia.

Diet plays a central role in glucose management, yet many individuals with T1D face challenges related to insulin dosing for specific meals.^6 7^ Limited data exist on which foods or meals are perceived as most problematic. Understanding these dietary challenges is essential for designing targeted healthcare strategies and education to support individuals with T1D.

Current dietary guidelines for adults with diabetes suggest that a range of macronutrient distributions can be effective for glucose control.^8 9^ Observational studies^10^,^12^ and small clinical trials^13^,^16^ in individuals with T1D have reported associations between reduced carbohydrate intake and improved glycaemic outcomes, while also noting potential negative effects on diet quality and lipid status when fat intake increases.^17 18^ Recent evidence supports a moderate carbohydrate intake (30% of energy, E%) as beneficial for glucose control.^19^ Carbohydrates have the greatest immediate impact on postprandial glucose levels, making management especially challenging. Fat and protein also affect glucose levels and require consideration in meal planning.^20^ Moreover, carbohydrate quality (eg, fibre content and glycaemic index) impacts glycaemic response,^21^,^23^ while fat quality (saturated vs unsaturated) may influence lipid metabolism.^24^

Despite the central role of diet in diabetes management, data on the relationship between dietary intake and glucose metrics in individuals with T1D remain limited. Addressing this gap is critical for advancing knowledge of diet-related challenges and improving clinical support for this population.

The primary aim of this study was to explore dietary intake and perceived challenges related to glucose management among individuals with T1D, with a focus on specific foods and mealtime situations. A secondary aim was to explore associations between dietary intake and participants’ clinical and demographic characteristics.

## Methods

### Study design and participants

This was a cross-sectional study of observational data collected through a digital survey. Participants were recruited from three diabetes specialist centres in Sweden. Using a digital random number generator, individuals were randomly selected from patient lists at each centre. Inclusion criteria included adults aged 18–75 years with T1D and diabetes duration of more than 1 year to ensure that participants had reached a more stable treatment phase. Exclusion criteria were inability to provide accurate responses due to factors such as language barriers or cognitive impairments. The upper age limit was set due to the use of digital questionnaires, as individuals above 75 years might face difficulties with the required technology.

Eligible individuals received an invitation letter detailing the study and were required to provide written informed consent before receiving login credentials for the survey. Study personnel followed up by phone with individuals who did not respond to assess their interest in participating. On obtaining consent, participants were provided with login details for the electronic survey. To maximise participation, follow-up reminders were sent to those who had not completed the survey. Study personnel also reached out to offer assistance or clarify any issues that might have prevented participants from completing the survey.

### Data collection

The electronic survey consisted of two questionnaires about diet. Dietary intake and habits were primarily assessed using the validated, semiquantitative food frequency questionnaire (FFQ), Meal-Q.^25^ This questionnaire includes 174 items on foods, meals and drinks, as well as questions on dietary habits, reflecting dietary intake over the past few months (<3 months). Respondents chose from predefined food items and intake frequencies, reporting only those they consumed at least once a month. For each of the following food groups, five photos of portion sizes were included: (1) rice, potatoes and pasta; (2) meat, chicken, fish and vegetarian substitutes and (3) vegetables (raw or cooked). The photos were used to calculate portion sizes for cooked dishes and vegetables. For other food items, standard portion sizes were used based on information from the National Food Agency. Nutrient averages were calculated from the responses using the national database on nutrient content (Livsmedelsdatabasen) administered by the Swedish National Food Agency (Livsmedelsverket). Macronutrient intakes were expressed both in absolute amounts (g/day) and as the percentage of total energy intake (E%).

A second questionnaire, developed by the research team consisting of dietitians, physicians, diabetes nurses and patient representatives, addressed additional topics not covered by Meal-Q. Designed to provide deeper insight into diet-related challenges in T1D, the questionnaire focused on carbohydrate counting, dietary management difficulties and contact with dietitians, covering dietary habits, support needs, challenges with carbohydrate-rich foods, postprandial glucose control and meal patterns. It included yes/no options, multiple-choice responses, open-ended questions and Likert scale ratings. The full questionnaire is available in the online supplemental material.

Estimated time to complete both questionnaires was approximately 30 min. Participants had the option of completing them in one session or multiple sessions. Data collection occurred from August 2019 to August 2023.

Clinical and demographic data, including age, sex, physical activity levels, medications and diabetes-related complications, were retrieved from the Swedish National Diabetes Register (NDR). For each participant, the registry value closest to the survey completion date was selected to ensure temporal alignment between survey responses and NDR data. Additionally, information on educational level was obtained from the study-specific questionnaire.

A comparison was conducted between study participants and the NDR, which included approximately 49 000 adults with T1D from all diabetes outpatient clinics across Sweden. Additionally, a comparison with only those from the three participating study sites was performed. Data from publicly available NDR reports were used.^26^ The 2021 data set was selected as best aligned with the survey period, providing the most relevant comparison.

### Statistical analyses

The required sample size was determined based on a previous study conducted by the research group using similar methods.^27^ A target of 500 participants was set, assuming a 70% response rate, resulting in an expected sample size of 350. To ensure the feasibility of follow-up and improve the response rate among those contacted, invitations to the survey were ultimately distributed to 410 individuals instead of the initially targeted 500.

Descriptive data are presented as mean (SD), median (IQR) or range (minimum and maximum value) for continuous variables. Numbers and percentages are presented as categorical variables.

Correlation analyses were conducted using the Pearson correlation coefficients to explore associations between participant characteristics such as age, sex, glycated haemoglobin A1c (HbA1c), insulin delivery method, physical activity level, educational level, mean glucose, SD of glucose values, percent time in range (%TIR, 3.9–10 mmol/L) and the following variables: mean energy intake (kcal), carbohydrates (grams/E%), fat (grams/E%), protein (grams/E%), fibre (grams/E%), whole grain (grams), monosaturated, polyunsaturated and saturated fat (grams/E%) and carbohydrate counting usage.

Differences between sexes (female vs male) and routes of insulin delivery (pump vs multiple daily injection (MDI)) were evaluated using independent samples *t*-test with corresponding 95% CIs.

All statistical tests were two-sided and conducted at the 5% significance level. Analyses were performed using IBM SPSS Statistics for Windows, V.28.0 (IBM Corp, Armonk, NY, USA) and SAS/STAT Software, V.9.4 (SAS Institute Inc., Cary, NC, USA).

### Patient and public involvement

Patients and members of the public were not directly involved in the study design or conduct of the study. However, when designing the study and developing the second questionnaire including questions about diabetes-related challenges, we discussed their relevancy with people either living with T1D themselves or having family members with T1D.

## Results

### Study participants

A total of 410 adults with T1D were randomly selected from patient lists across the three study sites. Of these, 35 individuals were excluded due to relocation, protected identity, incorrect diagnosis or inability to answer the questions or not fulfilling inclusion criteria (see online supplemental Figure S1 for the study participation flow chart). Ultimately, 191/375 (51%) participants met the inclusion criteria and consented to participate.

The mean (SD) age of participants was 48 (16) years, HbA1c was 56 (13) mmol/mol or 7.3 (1.1) %, mean glucose was 8.8 (2.2) mmol/L and TIR was 64% (18). The mean (SD) duration of diabetes was 28 (16) years, body mass index (BMI) was 27 (4.3) kg/m^2^ and 48% of participants were female. Insulin pumps were used by 41%, of whom 70% were female. Full participant characteristics are presented in table 1.

**Table 1:** Characteristics of the study population (n=191)

	Mean (SD) or n/N (%)
Age (years)	48 (16)
Female sex	92 (48.4%)
Body mass index (kg/m^2^)	27 (4.3)
Smoker, n/N (%)	11/170 (6.5%)
Diabetes duration (years)	28 (16)
**Glycaemic control and glucose metrics**	
HbA1c (mmol/mol)	56.0 (12.5)
HbA1c (%)	7.3 (1.1)
Mean glucose (mmol/L)	8.8 (2.2)
SD of glucose values (mmol/L)	3.0 (0.8)
Time in range (% of time 3.9–10 mmol/L)	63.7 (17.9)
**Blood pressure and lipid profile**	
Systolic blood pressure (mm Hg)	126.4 (12.3)
Diastolic blood pressure (mm Hg)	71.2 (9.7)
Use of antihypertensive drugs, n/N (%)	71/182 (39.0%)
Total cholesterol (mmol/L)	4.2 (0.75)
Low-density lipoprotein (mmol/L)	2.3 (0.66)
High-density lipoprotein (mmol/L)	1.6 (0.43)
Triglycerides (mmol/L)	0.96 (0.65)
Use of lipid lowering drugs, n/N (%)	91/182 (50.0%)
Creatinine (µmol/L)	78 (33)
Urine albumin to creatinine ratio (mg/mmol)	4.3 (20.3)
**Insulin therapy and dietary management**	
Route of insulin delivery, n/N (%)	
Multiple daily injections	107/181 (59.1%)
Insulin pump	74/181 (40.9%)
Carbohydrate counting usage, n/N (%)	27/116 (23.3%)
**Educational level, n/N (%)**	
Not completed primary school	1/164 (0.6%)
Primary school or equivalent	12/164 (7.3%)
Secondary school or equivalent	83/164 (50.6%)
University degree or equivalent	68/164 (41.5%)
**Physical activity level, n/N (%)**	
None	7/167 (4.2%)
<1 time/week	8/167 (4.8%)
Regularly 1–2 times/week	28/167 (16.8%)
Regularly 3–5 times/week	67/167 (40.1%)
Daily	57/167 (34.1%)
**Diabetes-related complications and comorbidities**	
Diabetes retinopathy, n/N (%)	132/179 (73.7%)
Treated for eye complications due to diabetes during the last year, n/N (%)	7/129 (5.4%)
Severe hypoglycaemia in the last 12 months, n/N (%)	1/176 (0.6%)
Ischaemic heart disease, n/N (%)	12/181 (6.6%)
Cerebrovascular disease, n/N (%)	7/181 (3.9%)

Descriptive data are presented as mean (SD) for numeric variables and as counts, number of responders and percentage of responders for categorical variables.

HbA1c, glycated haemoglobin A1c; SD, standard deviation.

### Dietary intake among participants measured by food frequency questionnaire

**Figure 1:**
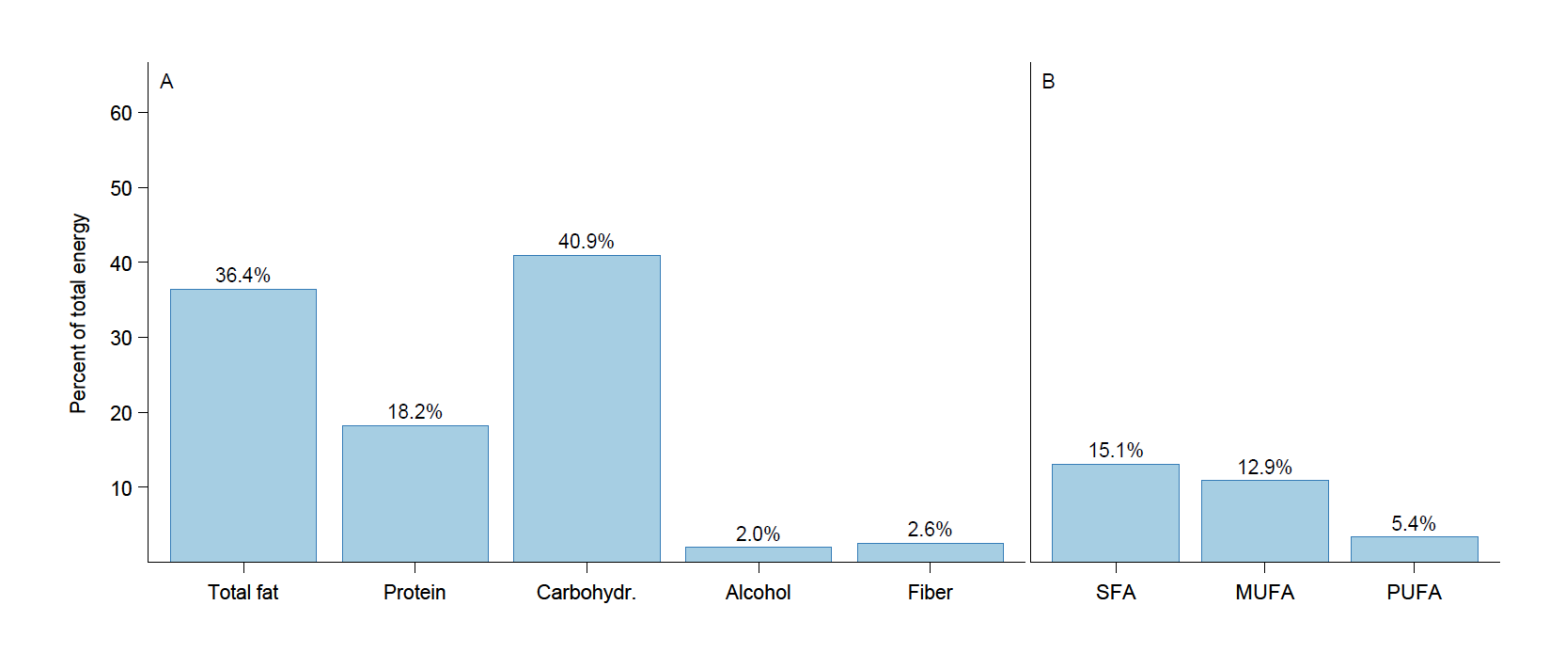
Distribution of macronutrients. (**A**) Proportion of total energy intake (%) derived from macronutrients, including fat, protein, carbohydrates, alcohol and fibre. (**B**) Composition of fat intake, showing the percentage of total energy from saturated (SFA), monounsaturated (MUFA) and polyunsaturated (PUFA) fatty acids. MUFA, monounsaturated fatty acids; PUFA, polyunsaturated fatty acids; SFA, saturated fatty acids.

Mean and median dietary intake of energy and macronutrients are shown in figure 1 and online supplemental Table S1. Mean (SD) energy intake was 1770 (750) kcal/day. Mean (SD) carbohydrate intake was 183 (91) g, contributing 41% (7) of energy (E%). Fibre and whole grain intake averaged 23 (15) g and 65 (67) g, respectively. Mean fat intake was 72 (32) g, contributing 36 (6) E%, while mean protein intake was 80 (32) g, accounting for 18 (3) E%. Mean saturated fatty acid (SFA) intake was 29 (14) g, representing 15 (3) E%.

### Dietary habits and diabetes-related difficulties

When asked about dietary patterns, 59% reported following the Swedish general dietary guidelines (National Food Agency), 14% adhered to diabetes-specific dietary guidelines, 8% followed a Mediterranean diet, 6% followed a vegetarian diet, 6% adhered to a low carbohydrate diet and 7% reported following other types of diets (table 2).

**Table 2:** Dietary habits, perceptions and self-management among study participants

	% of responders
**What type of diet do you usually follow?** (n=160)	
According to the Swedish Food Agency’s public recommendations	59.4%
Diabetes diet (wholegrains, foods with low glycemic index, vegetables, legumes, nuts, fish, lean meat, oils)	13.8%
Mediterranean diet (a lot of vegetables, olive oil, nuts, fish, lean meat)	8.1%
Vegetarian diet (plant-based foods including milk and/or eggs)	5.6%
Vegan diet (only plant-based foods)	0.0%
Low carbohydrate diet (less carbohydrates, more protein and fat)	5.6%
Extreme low carbohydrate diet (almost no carbohydrates and a lot of fat and protein)	0.6%
Other diet	6.9%
**How important do you consider diet to be for maintaining long-term glucose control?** (n=163)	
Not at all important	0.0%
Not particularly important	0.0%
Fairly important	11.6%
Very important	54.0%
Extremely important	31.9%
Do not know/no opinion	2.5%
**Are you currently following a diet that you believe is the best for you?** **(to feel well and maintain glucose control)** (n=163)	
Does not apply at all	2.5%
Partially applies	23.3%
Neither applies nor does not apply	14.7%
Largely applies	50.3%
Fully applies	8.0%
Do not know/no opinion	1.2%
**Do you believe that you could achieve significantly better glucose levels by changing how/what you eat?**(n=162)	
Does not apply at all	12.9%
Partially applies	34.6%
Neither applies nor does not apply	11.1%
Largely applies	28.4%
Fully applies	10.5%
Do not know/no opinion	2.5%
**Do you consciously reduce the amount of carbohydrates at your meals to achieve better glucose control?** (n=159)	
Does not apply at all	12.7%
Partially applies	27.7%
Neither applies nor does not apply	10.2%
Largely applies	33.7%
Fully applies	14.5%
Do not know/no opinion	0.6%
**Do you usually choose whole grain products regarding bread/pasta/flour?** (n=163)	
Does not apply at all	11.0%
Partially applies	28.2%
Neither applies nor does not apply	8.0%
Largely applies	33.7%
Fully applies	17.2%
Do not know/no opinion	1.8%

There were 58% of participants who reported following a diet that they percieved as most beneficial for their well-being and for maintaining glucose control. Additionally, 39% believed that changing their eating habits could help them achieve significantly better glucose control (table 2).

**Figure 2:**
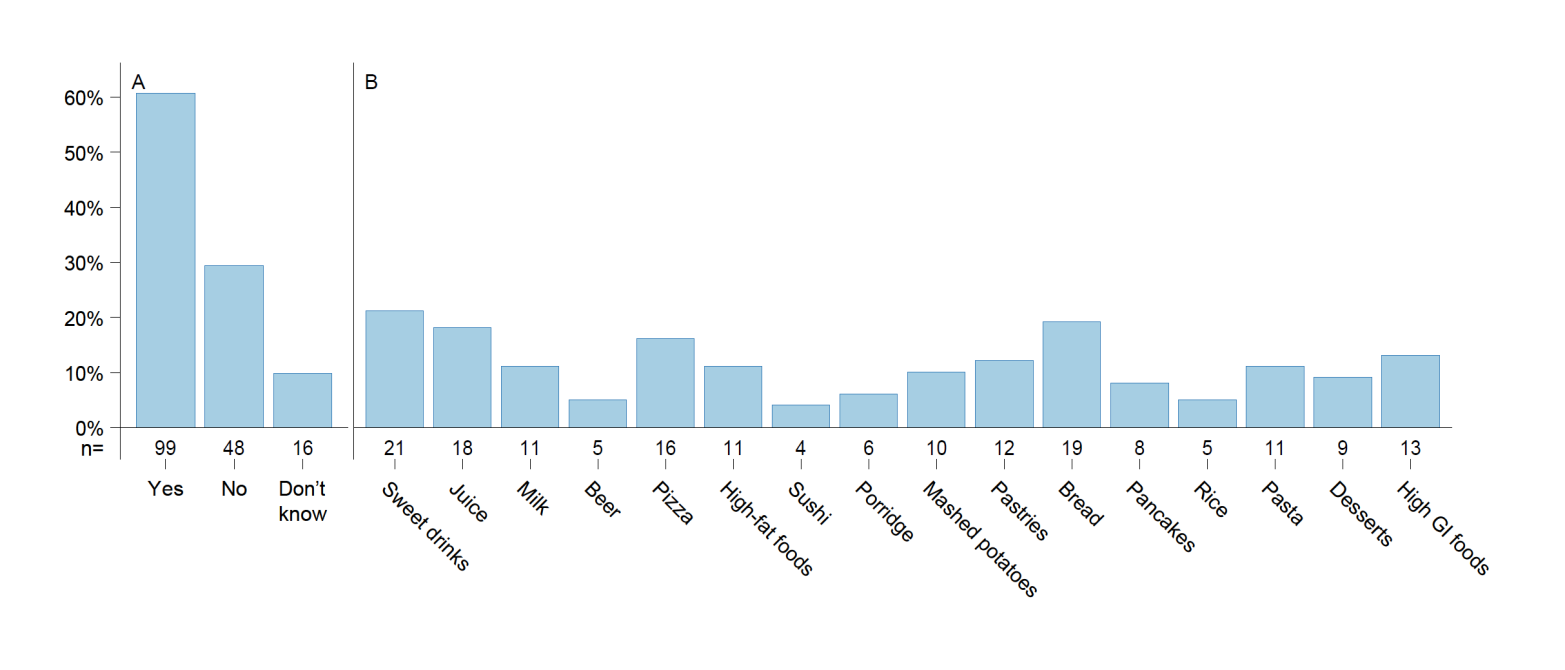
Avoided foods/meals due to diabetes. (**A**) Is there any food or dish that you avoid or eat/drink less often because it negatively affects your glucose control due to diabetes but that you wish you could eat/drink more of? (**B**) Examples of commonly avoided foods.

Half of the participants reported that they most often choose whole grain products (bread, pasta, flour) (table 2), and more participants indicated that white bread posed greater challenges in managing insulin doses compared with whole grain bread (online supplemental Table S2). There were 61% of participants that reported avoiding certain types of foods due to difficulties associated with diabetes management. Commonly avoided foods and dishes included pizza, fried and high-fat foods, sweet beverages, juice, desserts, candy, mashed potatoes and porridge (figure 2). Nearly half of the participants (48%) reported consciously reducing carbohydrates in meals to achieve better glucose control (table 2). Additionally, factors such as carbohydrate amount and type, physical activity before or after meals, premeal glucose levels and other influences like stress, infection and insulin dose were considered important for maintaining postprandial glucose control (online supplemental Table S3 and S4).

Approximately 90% of participants reported having breakfast, lunch and dinner daily. Twelve percent had snacks three times per day, 46% consumed them 1–2 times per day, 21% a few times per week and 22% less frequently or never (online supplemental Table S5).

**Figure 3:**
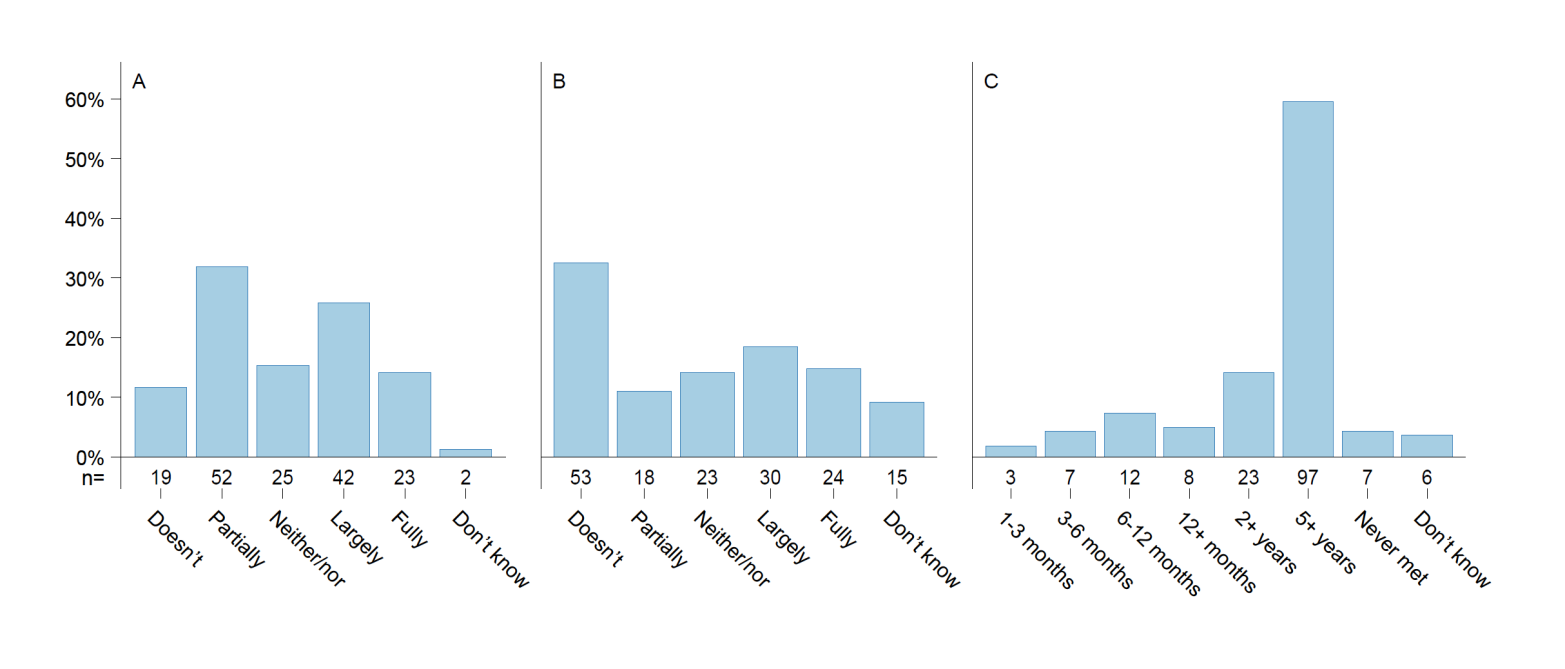
Need for dietary consultancy. (**A**) Do you feel that you have received sufficient information/help with your diet from healthcare (eg, diabetes nurse, doctor or dietitian) since being diagnosed with diabetes? (**B**) Do you currently wish to receive information/help with your diet from a dietitian? (**C**) How long has it been since you last received information/help with your diet from a dietitian?

There were 29% of participants that either did not use carbohydrate counting or were unaware of it. Among all participants, 53% relied on visual estimation to approximate carbohydrate content in meals, while 18% used more advanced methods. Despite this, 84% considered carbohydrate counting an important tool for achieving long-term glycaemic control, though 51% found it difficult to use (online supplemental Table S6). There were 40% of participants that reported that they had not received sufficient information or support with their diet from their healthcare providers since they got their diabetes diagnosis, and 33% expressed a wish for more help or information from a dietitian right now. Additionally, 60% reported that it had been over 5 years since their last appointment with a dietitian, while 4% reported never having had one (figure 3).

More than 50% of participants found it challenging to estimate the correct insulin dose in certain situations, including at parties with extended eating or alcohol consumption, dining at restaurants, managing low glucose levels or combining food with exercise (online supplemental Table S7).

All questions and results regarding dietary habits and diabetes-related difficulties are summarised in table 2 and in the online supplemental Tables S2–S7.

### Correlations between physical activity, BMI, sex and diet

Higher physical activity was associated with a higher intake of carbohydrates (g and E%; r=0.19, p=0.038 and p=0.036), fibre (r=0.25, p=0.004) and whole grains (r=0.26, p=0.004) and negatively associated with total fat (E%; r = –0.25, p=0.005), monounsaturated fatty acids (MUFA) (E%; r = –0.21, p=0.016) and SFA (E%; r = –0.31, p<0.001) (online supplemental Table S8). Higher BMI correlated with higher protein intake (E%; r=0.18, p=0.039) and lower polyunsaturated fat (E%; r = –0.22, p=0.012). Both higher HbA1c and mean glucose were linked to lower polyunsaturated fatty acids (PUFA) (E%; r = –0.19, p=0.030 and r = –0.20, p=0.042), whereas greater TIR was associated with higher intake of PUFA (r=0.27, p=0.006), lower carbohydrate intake (E%; r = –0.24, p=0.018) and a tendency toward increased use of carbohydrate counting (r=0.17, p=0.056). Females had lower protein intake (73 (27) g vs 86 (35) g, p=0.018) and higher fibre (E%; 2.7 (0.9) vs 2.4 (0.8), p=0.020) and PUFA (E%; 5.8 (1.9) vs 4.9 (1.6), p=0.003) (online supplemental Table S9).

### Other variables related to dietary habits

Insulin pump users more often applied carbohydrate counting: 53% (n=39/73) compared with 25% (n=24/98) participants using MDI (online supplemental Table S10) p<.001). Pump usage was also associated with more TIR (68% vs 61%, p=0.026) and with lower SD of glucose values (2.8 (0.7) vs 3.2 (0.8), p=0.011). A higher degree of education was also associated with greater intake of polyunsaturated fats in E% (r=0.24, p=0.005) and an increased use of carbohydrate counting: 17% (n=2/12) among those completing primary school, 35% (n=29/83) of those with secondary school education and 50% (n=34/68) among those with a university degree (p=0.011). No significant associations were observed between age and dietary variables or questionnaire responses (online supplemental Table S8).

### Comparison of study participants to national and local type 1 diabetes populations

Compared with the general adult population with T1D in Sweden in 2021,^26^ study participants included a slightly higher proportion of females (48% vs 44%), had a slightly lower mean HbA1c level (56 vs 59 mmol/mol) and more frequently used insulin pumps (41% vs 28%). Comparisons with the local NDR data from the three study sites (NU Hospital Group, Uppsala University Hospital and Vrinnevi Hospital in Norrköping) showed similar differences. Other characteristics, including age, diabetes duration, BMI and blood pressure levels, were comparable with both the general adult T1D population and the general populations from study sites (online supplemental Table S11).

## Discussion

This Swedish diet survey reveals that fewer than one in five adults with T1D used advanced carbohydrate counting, despite most participants acknowledging its overall importance for glycaemic control. Many found it challenging to apply. Mean carbohydrate intake was 41% of total energy, with many participants intentionally restricting carbohydrates and avoiding foods like sushi, pizza, sweets and sweet beverages. Participants favoured fibre-rich options, such as whole grain bread and pasta, which they found less challenging for glucose control compared with their refined counterparts. Fat and carbohydrate quality did not fully align with dietary recommendations.

Mean carbohydrate intake in this population was relatively low (41 E%) but consistent with other diabetes populations in Sweden^19 28^ and Australia^29^ of 41–42 E%. However, these data were derived from randomised or case-control studies rather than population-based samples, which may limit generalisability. Current dietary guidelines and reviews suggest that a wide range of carbohydrate intakes can be appropriate, provided that fibre and SFA targets are met.^8 30 31^ The sucrose intake was 6 E%, indicating that the intake of added sugar was in line with recommendations (<10 E%).^8^ To be noted, hypoglycaemia management often necessitates or induces intake of simple sugars (and sometimes foods rich in SFA like sweets, desserts and chocolate), which complicates dietary quality assessments in T1D populations.

Survey responses indicated that participants found carbohydrate counting and high-carbohydrate foods challenging, leading many to intentionally reduce intake. However, fibre-rich options like whole grain bread, pasta and rice were perceived as easier to manage for glucose control. A previous study from this group has linked moderate carbohydrate intake (30 E%) with improved glucose control and treatment satisfaction.^19^ Additionally, diets high in fibre^22^ or low in glycaemic index^21 23 32^ have been associated with better glucose regulation, supporting participants’ experiences of improved glucose control with more whole grain and fibre-rich foods.

Beyond carbohydrates, fat and protein also influence glucose levels.^20 33 34^ Fat delays gastric emptying, causing prolonged postprandial hyperglycaemia, while free fatty acids may contribute to insulin resistance. Protein intake can lead to delayed hyperglycaemia through gluconeogenesis and increased glucagon secretion.^33^ Participants reported difficulties managing high-fat, high-carbohydrate foods like pizza, likely due to difficulties in timing and dosing insulin for delayed glucose responses. Protein intake was not frequently mentioned as problematic, though this aspect was less explored in the survey.

Dietary adherence varies among individuals with diabetes compared with the general population, with some studies showing similar adherence^29^ and others indicating better adherence.^35^ In our study, participants exceeded the recommendations of <10% of energy from SFA and fell short of the ≥35 gram per day fibre target,^8^ mirroring trends in other diabetes populations.^19 29^ This is probably due to the relatively low carbohydrate and higher fat intake reported in the study. Given the association between high SFA intake and increased LDL cholesterol,^24^ as well as fibre’s benefits for glucose control and cardiovascular health,^22^ these findings highlight the need for improved dietary guidance in this population.

Advanced carbohydrate counting was uncommon, with 53% using visual estimation and only 18% using advanced techniques. It was primarily used by insulin pump users, likely due to integrated pump features and training during initiation. Despite its availability to all individuals with T1D in Sweden, interest remains low, especially among those using MDI who may not perceive its benefits. There is evidence for the effect on glucose control and treatment satisfaction with advanced carbohydrate counting^36^ even if the results from reviews and meta-analyses are heterogenous.^37^ A randomised trial comparing structured carbohydrate counting with diabetes-specific healthy eating education and a control group over 12 months showed no significant HbA1c difference between groups. However, participants in the healthy eating group improved their diet.^28^ Combining structured carbohydrate counting with education on healthy food choices might yield more impactful results on HbA1c and other health outcomes.

Higher HbA1c and mean glucose levels were associated with lower PUFA intake, while greater TIR was associated with higher PUFA and lower carbohydrate intake. More physical activity was associated with greater adherence to dietary guidelines. This could possibly be due to increased health awareness.^38^ For some individuals, lower carbohydrate intake might be a sign of health awareness, and for those exercising more, it could be more important to increase carbohydrates for maintaining glycaemic control and glycogen storage.^39^

The results from this survey have several implications. Many individuals living with T1D appear to experience challenges related to diet and glucose management. Approximately 40% of participants reported not having received sufficient dietary support from healthcare providers since their diagnosis, and approximately 30% expressed a current desire for consultation with a dietitian, highlighting an area for improvement in routine care. Participants’ dietary intake did not align to dietary guidelines regarding fibre and saturated fat intake, further underscoring the potential value of dietitian-led support. Additionally, 40% of participants believed that dietary changes could improve their glucose control. While many participants expressed a desire for more dietitian support, previous research suggests that both healthcare system limitations and patient-related factors may contribute to low engagement. Barriers such as limited availability of dietitians, unclear referral processes and lack of structured follow-up have been identified within diabetes care.^40^ Additionally, some individuals may perceive dietary counselling as less relevant or face practical challenges such as time constraints or difficulties accessing services.^41^ Providing comprehensive education on both carbohydrate counting and general dietary principles may help alleviate dietary challenges and improve overall diet quality among individuals with T1D.

### Strengths and limitations

Key strengths of this study include the random selection of participants and multicentre design, thus enhancing representativeness and reducing selection bias. However, recruitment challenges led to a lower-than-expected response rate (51% vs 70% targeted). While lower than anticipated, this rate is relatively high compared with similar surveys.^42^ Recruitment barriers included challenges in reaching participants and incomplete survey submissions despite multiple reminders. Although reminders likely improved participation, a shorter questionnaire might have further increased the response rate.^42 43^ To assess generalisability, participant characteristics were compared with data from the Swedish NDR for the general adult population with T1D in 2021.^26^ While most characteristics were similar, participants in this study had slightly lower HbA1c, more used insulin pumps and a slightly higher proportion were females. This may reflect greater health interest among these individuals,^38^ making them more likely to participate in a dietary survey. Additionally, pump users tend to have lower HbA1c than MDI users,^44 45^ which was also reflected in our findings, where pump users showed lower glucose variability and higher TIR.

The use of a validated FFQ strengthened the reliability of dietary assessment. However, self-reported intake introduces potential recall bias and misreporting, which is common in dietary studies.^46 47^ The relatively low mean energy intake suggests possible under-reporting, a known limitation of all dietary assessment methods.^48^ Nevertheless, since our primary focus was on diet composition (macronutrient distribution, E%) rather than absolute intake, no further adjustments were made. The second questionnaire was not previously validated, which may limit its reliability. As these analyses were hypothesis-generating, no adjustments were made for multiple testing, increasing the risk of type I errors. Therefore, results should be interpreted with caution. Lastly, most participants were using either conventional insulin pumps or MDI at the time of the study. Although the use of automated insulin delivery systems is increasing in high-income countries, MDI and conventional pumps remain the most commonly used treatment modalities globally.

## Conclusions

This population of Swedish adults with T1D reported lower fibre and whole grain intake and higher saturated fat intake compared with dietary guidelines. Advanced carbohydrate counting methods were not widely used and were perceived as challenging to apply. Many participants intentionally limited carbohydrate intake, with some avoiding certain carbohydrate-rich foods due to difficulties in glucose management. Approximately 40% of participants believed that dietary changes could improve glucose control, and a similar proportion reported insufficient dietary guidance from healthcare professionals since diagnosis. About one-third expressed a desire for additional support from dietitians. These findings indicate a need to strengthen access to tailored nutritional support within routine diabetes care, with a focus on practical strategies for meal planning and carbohydrate management.

## Supplementary material

10.1136/bmjopen-2025-101619 online supplemental file 1

10.1136/bmjopen-2025-101619 online supplemental file 2

## Data Availability

Data are available upon reasonable request.
