# Supplementary material for: Diet and diet-related challenges with specific focus on carbohydrates and carbohydrate counting in adults with type 1 diabetes: a cross-sectional study
Source: BMJ Open. 2025 Nov 29;15(11):e101619. doi: 10.1136/bmjopen-2025-101619 (PMC12666139; doi:10.1136/bmjopen-2025-101619)
Supplement: online supplemental file 1 [file bmjopen-15-11-s001.docx]

This supplement has been provided by the authors to provide readers with additional information about their work. Supplement to

**Diet and diet related challenges with specific focus on carbohydrates and carbohydrate counting in adults with type 1 diabetes: a cross-sectional study**

Authors: Sofia Sterner Isaksson^1,2^, Jarl Hellman^3^, Magnus Wijkman^4^, Henrik Imberg^1,5^, Arndís. F. Ólafsdóttir^1^, Mette Axelsen^6,7^, Marcus Lind^1,2,8^

Affiliations:

1. Department of Molecular and Clinical Medicine, Institute of Medicine, Sahlgrenska Academy, University of Gothenburg, Gothenburg, Sweden
2. Department of Medicine, NU Hospital Group, Uddevalla, Sweden.
3. Uppsala University, Department of Medical Sciences, Uppsala, Sweden
4. Department of Internal Medicine and Department of Health, Medicine and Caring Sciences, Linköping University, Norrköping, Sweden
5. Statistiska Konsultgruppen Sweden, Gothenburg, Sweden
6. Institute of Medicine, Sahlgrenska Academy, University of Gothenburg, Gothenburg, Sweden
7. Global Oatly Science and Innovation Centre, Lund, Sweden
8. Department of Medicine, Geriatrics and emergency care, Sahlgrenska University Hospital, Gothenburg, Västra Götaland, Sweden

**Table of contents**

**Table S1.** Distribution of macronutrients recorded using Meal-Q…..………………………………………3

**Table S2.** Challenges with carbohydrate-rich foods/meals………………………………………………….4

**Table S3.** Dietary and other factors in relation to experienced challenges with low postprandial glucose levels……………………………………………………………………………………………………………....5

**Table S4.** Dietary and other factors in relation to experienced challenges with high postprandial glucose levels…………………………………………………………………………………………………….6

**Table S5.** Frequency of meal and snack consumption………………………………………………………7

**Table S6.** Questions regarding carbohydrate counting………………………………………………………8

**Table S7.** Challenges at different meals and occasions……………………………………………………..9

**Table S8.** Correlations between dietary variables and participant characteristics………………………10

**Table S9.** Differences in dietary and glucose variables by sex.………………………………………...…11

**Table S10.** Differences in dietary variables by pump usage.………………………………………………12

**Table S11.** Comparison of study participant characteristics with the Swedish NDR 2021……………..13

**Figure S1.** Flow chart of study participation………………………………………………………………...14

**Supplemental Table S1.** Distribution of macronutrients recorded using Meal-Q (n=136).

|  | Mean (SD) | Median (Q1–Q3) |
| --- | --- | --- |
| Energy intake (kcal) | 1769 (751) | 1624 (1268–2102) |
| Protein (g) | 80 (32) | 75 (56–94) |
| Protein (E%) | 18 (3) | 18 (17–20) |
| Total fat (g) | 72 (32) | 64 (49–87) |
| Total fat (E%) | 36 (6) | 36 (33–41) |
| Saturated fatty acids (g) | 29 (14) | 26 (20–36) |
| Saturated fatty acids (E%) | 15 (3) | 15 (13–17) |
| Monounsaturated fatty acids (g) | 25 (11) | 23 (17–31) |
| Monounsaturated fatty acids (E%) | 13 (2) | 13 (11–15) |
| Polyunsaturated fatty acids (g) | 11 (6) | 10 (6–14) |
| Polyunsaturated fatty acids (E%) | 5 (2) | 5 (4–7) |
| Carbohydrates (g) | 183 (91) | 161 (128–217) |
| Carbohydrates (E%) | 41 (7) | 41 (37–46) |
| Fiber (g) | 23 (15) | 21 (14–29) |
| Fiber (g/MJ) | 3.1 (0.1) | 3.1 (2.0–4.3) |
| Alcohol (g) | 5 (5) | 3 (1–8) |
| Alcohol (E%) | 2 (2) | 1 (0–3 |
| Sucrose (g)  Sucrose (E%) | 26 (19)  5.9 (4.3) | 22 (16–31)  5.4 (3.9-7.6) |
| Wholegrain (g) | 65 (67) | 50 (25–83) |
| Cholesterol (g) | 255 (117) | 241 (174–308) |
| Trans fat (g) | 1 (1) | 1 (1–1) |
| Monosaccharides (g) | 28 (16) | 23 (19–34) |
| Disaccharides (g) | 54 (26) | 50 (37–64) |
| **Abbreviations**: E%, percent of total energy intake; g/MJ; gram per Mega Joule, SD, standard deviation; Q1, lower quartile; Q3, upper quartile. | | |

**Supplemental Table S2.** Challenges with carbohydrate-rich foods/meals (n=160).

How easy or difficult do you find it to choose the correct insulin dose for a meal and achieve your target glucose range (1.5–2 hours after the meal) when eating the following carbohydrate-rich foods/dishes?

|  | Very easy | Easy | Fairly easy | Neither easy nor difficult | Fairly difficult | Difficult | Very difficult | Don’t know/no opinion |
| --- | --- | --- | --- | --- | --- | --- | --- | --- |
| Pasta | 1.8 | 10.8 | 18.7 | 20.5 | 18.1 | 13.3 | 5.4 | 10.2 |
| Pasta (whole grain) | 3.6 | 10.8 | 18.7 | 17.5 | 13.3 | 6.6 | 1.8 | 26.5 |
| Rice | 2.4 | 9.6 | 21.1 | 22.3 | 15.1 | 9.6 | 6.6 | 11.4 |
| Rice (whole grain) | 3.6 | 7.8 | 18.7 | 16.3 | 12.7 | 4.8 | 1.8 | 33.1 |
| Cooked grains such as bulgur, wheat berries, and barley (used instead of rice) | 3.0 | 9.0 | 16.3 | 14.5 | 12.0 | 4.8 | 1.2 | 38.0 |
| White bread | 1.2 | 9.0 | 13.3 | 14.5 | 19.3 | 16.9 | 12.7 | 12.0 |
| Bread (medium fiber/wholegrain content) | 1.8 | 14.5 | 21.7 | 19.3 | 13.3 | 9.6 | 4.2 | 14.5 |
| Whole grain bread (like rye bread) | 7.2 | 15.7 | 25.3 | 19.9 | 9.6 | 3.6 | 1.8 | 16.3 |
| Bread with sour dough | 4.8 | 10.8 | 20.5 | 24.7 | 7.2 | 5.4 | 3.6 | 21.1 |
| Pizza | 2.4 | 4.2 | 8.4 | 12.7 | 18.1 | 19.9 | 24.7 | 8.4 |
| Boiled potatoes | 7.2 | 17.5 | 21.1 | 22.9 | 14.5 | 5.4 | 3.6 | 6.6 |
| Fried/deep fried potatoes | 1.8 | 9.6 | 16.3 | 21.1 | 18.7 | 14.5 | 6.6 | 10.2 |
| Mashed potatoes | 1.8 | 8.4 | 10.8 | 15.1 | 24.7 | 16.3 | 13.3 | 8.4 |
| Row percentages are presented. | | | | | | | | |

**Supplemental Table S3.** Dietary and other factors in relation to experienced challenges with low postprandial glucose levels (n=160).

To prevent post-meal hypoglycemia (low glucose levels), how important do you consider the following factors?

|  | Not at all important | Not particularly important | Fairly important | Very important | Extremely important | Don’t know/no opinion |
| --- | --- | --- | --- | --- | --- | --- |
| Amount of carbohydrates in the meal (e.g., bread, rice, pasta, potatoes, sugar etc.) | 2.4 | 4.8 | 25.3 | 38.0 | 22.9 | 6.0 |
| Type of carbohydrates (e.g., fast acting, slow) | 1.2 | 4.8 | 28.3 | 34.9 | 22.9 | 7.2 |
| If the meal contains a lot of fat | 3.6 | 9.3 | 29.5 | 26.5 | 9.6 | 10.8 |
| If the meal contains a lot of protein (e.g., meat, fish, eggs) | 8.4 | 42.2 | 24.1 | 11.4 | 3.6 | 9.6 |
| If the meal contains a lot of fiber (e.g., whole grains, flax seeds etc.) | 3.0 | 18.1 | 37.3 | 22.9 | 6.0 | 12.0 |
| Amount of insulin provided to the meal | 0.6 | 4.8 | 27.1 | 65.1 | 0 | 1.8 |
| Type of bolus insulin taken to the meal (e.g., split dose or extended dose) | 1.2 | 7.8 | 16.3 | 16.9 | 18.1 | 38.6 |
| Timing of insulin dosing for the meal | 1.2 | 6.6 | 25.3 | 9.8 | 22.3 | 4.2 |
| Physical activity/exercise before the meal | 0 | 3.6 | 19.9 | 33.1 | 32.5 | 10.2 |
| If you plan to exercise after the meal | 1.2 | 3.0 | 12.7 | 30.7 | 44.6 | 7.2 |
| If you have checked your glucose levels before meal | 0.6 | 4.2 | 9.6 | 30.7 | 50.0 | 7 4.2 |
| How you feel around mealtime (e.g., stress, infection etc.) | 4.2 | 10.8 | 18.1 | 21.7 | 33.1 | 11.4 |
| Row percentages are presented. | | | | | | |

**Supplemental Table S4.** Dietary and other factors in relation to experienced challenges with high postprandial glucose levels (n=160).

To prevent post-meal hyperglycemia (high glucose levels), how important do you consider the following factors?

|  | Not at all important | Not particularly important | Fairly important | Very important | Extremely important | Don’t know/no opinion |
| --- | --- | --- | --- | --- | --- | --- |
| Amount of carbohydrates in the meal (e.g., bread, rice, pasta, potatoes, sugar etc.) | 0 | 1.8 | 13.9 | 41.0 | 41.0 | 1.8 |
| Type of carbohydrates (e.g., fast acting, slow) | 0.6 | 0.6 | 18.7 | 39.2 | 38.0 | 2.4 |
| If the meal contains a lot of fat | 5.4 | 22.3 | 27.7 | 24.1 | 12.0 | 7.8 |
| It the meal contains a lot of protein (e.g., meat, fish, eggs) | 9.6 | 38.6 | 24.1 | 15.1 | 3.0 | 9.0 |
| It the meal contains a lot of fiber (e.g., whole grains, flax seeds etc.) | 5.4 | 18.7 | 35.5 | 22.9 | 6.0 | 10.8 |
| Amount of insulin provided to the meal | 0.6 | 0 | 7.8 | 25.9 | 63.9 | 1.2 |
| Type of bolus insulin taken to the meal (e.g., split dose or extended dose) | 0.6 | 6.0 | 12.0 | 20.5 | 21.1 | 38.6 |
| Timing of insulin dosing for the meal | 0.6 | 3.6 | 21.1 | 38.0 | 33.7 | 2.4 |
| Physical activity/exercise before the meal | 0.6 | 6.6 | 30.1 | 27.7 | 27.1 | 7.2 |
| If you plan to exercise after the meal | 1.2 | 8.4 | 19.9 | 30.1 | 33.7 | 6.0 |
| If you have checked your glucose levels before meal | 0.6 | 1.8 | 14.5 | 31.3 | 48.8 | 2.4 |
| How you feel around mealtime (e.g., stress, infection etc.) | 1.8 | 9.0 | 21.1 | 30.1 | 28.3 | 9.0 |
| Row percentages are presented. | | | | | | |

**Supplemental Table S5.** Frequency of meal and snack consumption according to Meal-Q (n=136).

| How often do you usually eat or drink something at the following meals? | % of responders |
| --- | --- |
| Breakfast |  |
| Every day | 86.8% |
| Several times per week | 5.9% |
| Once per week | 1.5% |
| Less often or never | 5.9% |
| Lunch |  |
| Every day | 87.5% |
| Several times per week | 9.6% |
| Once per week | 0.7% |
| Less often or never | 2.2% |
| Dinner |  |
| Every day | 91.9% |
| Several times per week | 3.7% |
| Once per week | 3.7% |
| Less often or never | 0.7% |
|  |  |
| How often do you have a snack? |  |
| 3 times per day | 11.8% |
| 1–2 times per day | 45.6% |
| A few times per week | 20.6% |
| Less often or never | 22.1% |

**Supplemental Table S6.** Questions regarding carbohydrate counting.

|  | % of responders |
| --- | --- |
| **Do you use carbohydrate counting? (n=160)** |  |
| Do not use carbohydrate counting or do not know what it is | 29.4% |
| Estimates the amount of carbohydrates in the meal visually | 52.5% |
| Calculates the amount of carbohydrates in the meal by regularly weighing at least certain types of food included in the meals | 1.3% |
| Checks nutritional labels to calculate carbohydrate amounts in meals | 6.9% |
| Uses apps/websites/books to calculate carbohydrate amounts | 1.3% |
| Uses various mathematical rules such as the 100, 300, or 500 rule to determine the correct insulin-to-carbohydrate ratio | 1.3% |
| Uses the pump's carbohydrate counting functions to select the correct insulin dose | 7.5% |
|  |  |
| **How important do you consider carbohydrate counting to be for achieving long-term glucose control? (n=115)** |  |
| Not at all important | 0.9% |
| Not particularly important | 8.5% |
| Fairly important | 39.3% |
| Very important | 32.5% |
| Extremely important | 12.0% |
| Don’t know/no opinion | 6.8% |
|  |  |
| **How easy or difficult do you find carbohydrate counting to use? (n=105)** |  |
| Very easy | 3.8% |
| Easy | 1.9% |
| Fairly easy | 17.1% |
| Neither easy nor difficult | 26.7% |
| Fairly difficult | 34.3% |
| Difficult | 6.7% |
| Very difficult | 9.5% |
|  |  |
| **Have you received information or training on carbohydrate counting from healthcare providers (nurse, doctor, dietitian) in the past year? (n=152)** |  |
| Yes | 59.0% |
| No | 35.5% |
| Don’t know | 4.8% |

**Supplemental Table S7.** Challenges with different meals and occasions (n=160).

How easy or difficult do you find it to choose the correct insulin dose in these situations? (to achieve good glucose control approximately 1.5–2 hours afterward and beyond).

|  | Very easy | Easy | Fairly easy | Neither easy nor difficult | Fairly difficult | Difficult | Very difficult | Don’t know/no opinion |
| --- | --- | --- | --- | --- | --- | --- | --- | --- |
| Having breakfast | 10.8 | 19.9 | 28.9 | 10.8 | 15.7 | 6.0 | 6.0 | 1.2 |
| Having lunch | 6.0 | 17.5 | 32.5 | 18.1 | 6.9 | 4.8 | 1.8 | 1.8 |
| Having dinner | 4.8 | 16.9 | 31.3 | 21.1 | 15.1 | 5.4 | 3.1 | 1.8 |
| Having snacks | 6.0 | 15.1 | 23.5 | 22.3 | 10.8 | 5.4 | 1.8 | 13.9 |
| Having low glucose levels | 1.8 | 5.4 | 13.9 | 14.5 | 29.5 | 18.7 | 12.0 | 3.6 |
| At high glucose levels before a meal | 2.4 | 11.4 | 20.5 | 16.9 | 25.9 | 12.7 | 8.4 | 1.2 |
| At a party (extended eating, alcohol intake) | 2.4 | 1.8 | 8.4 | 11.4 | 34.3 | 21.1 | 12. | 6.6 |
| At a restaurant (not knowing exact content of meal) | 0 | 5.4 | 12.0 | 18.1 | 27.1 | 18.1 | 12.7 | 5.4 |
| In combination with exercise | 1.2 | 6.6 | 12.0 | 14.5 | 24.7 | 15.7 | 15.7 | 9.0 |
| Row percentages are presented. | | | | | | | | |

**Supplemental Table S8.** Correlations between dietary variables and participant characteristics (n=136).

|  | Participant characteristic | | | | | | |
| --- | --- | --- | --- | --- | --- | --- | --- |
| Dietary response | Age | Physical activity | BMI | HbA1c | Mean glucose | SD of glucose values | TIR |
| Total energy intake (kcal) | r=0.08 | r=0.15 | r=0.04 | r=−0.05 | r=0.03 | r=0.06 | r=−0.06 |
|  | *P*=0.35 | *P*=0.10 | *P*=0.68 | *P*=0.53 | *P*=0.78 | *P*=0.60 | *P*=0.54 |
|  |  |  |  |  |  |  |  |
| Carbohydrates (gram) | r=0.05 | r=0.19 | r=0.06 | r=−0.01 | r=0.06 | r=0.06 | r=−0.13 |
|  | *P*=0.57 | ***P*=0.038** | *P*=0.53 | *P*=0.88 | *P*=0.54 | *P*=0.59 | *P*=0.20 |
|  |  |  |  |  |  |  |  |
| Carbohydrates (E%) | r=−0.12 | r=0.19 | r=−0.05 | r=0.07 | r=0.10 | r=−0.03 | **r=−0.24** |
|  | *P*=0.15 | ***P*=0.036** | *P*=0.59 | *P*=0.41 | *P*=0.31 | *P*=0.79 | ***P*=0.018** |
|  |  |  |  |  |  |  |  |
| Fat (gram) | r=0.06 | r=0.06 | r=−0.02 | r=−0.09 | r=−0.00 | r=0.04 | r=0.01 |
|  | *P*=0.46 | *P*=0.54 | *P*=0.86 | *P*=0.28 | *P*=0.98 | *P*=0.71 | *P*=0.93 |
|  |  |  |  |  |  |  |  |
| Fat (E%) | r=0.01 | r=−0.25 | r=−0.06 | r=−0.08 | r=−0.07 | r=−0.00 | r=0.18 |
|  | *P*=0.88 | ***P*=0.005** | *P*=0.50 | *P*=0.36 | *P*=0.50 | *P*=0.97 | *P*=0.070 |
|  |  |  |  |  |  |  |  |
| Protein (gram) | r=0.12 | r=0.16 | r=0.07 | r=−0.09 | r=0.01 | r=0.07 | r=−0.02 |
|  | *P*=0.18 | *P*=0.077 | *P*=0.43 | *P*=0.32 | *P*=0.93 | *P*=0.53 | *P*=0.83 |
|  |  |  |  |  |  |  |  |
| Protein (E%) | r=0.13 | r=0.03 | **r=0.18** | r=−0.09 | r=−0.06 | r=0.11 | r=0.15 |
|  | *P*=0.13 | *P*=0.71 | ***P*=0.039** | *P*=0.31 | *P*=0.55 | *P*=0.31 | *P*=0.13 |
|  |  |  |  |  |  |  |  |
| Fiber (gram) | r=0.09 | r=0.25 | r=0.01 | r=−0.02 | r=−0.04 | r=0.02 | r=−0.01 |
|  | *P*=0.31 | ***P*=0.004** | *P*=0.87 | *P*=0.80 | *P*=0.72 | *P*=0.87 | *P*=0.88 |
|  |  |  |  |  |  |  |  |
| Fiber (E%) | r=−0.03 | r=0.31 | r=−0.15 | r=−0.01 | r=−0.14 | r=−0.11 | r=0.11 |
|  | *P*=0.77 | ***P*<.001** | *P*=0.081 | *P*=0.92 | *P*=0.15 | *P*=0.30 | *P*=0.27 |
|  |  |  |  |  |  |  |  |
| Wholegrain (gram) | r=0.13 | r=0.26 | r=0.05 | r=0.06 | r=0.03 | r=0.07 | r=−0.07 |
|  | *P*=0.14 | ***P*=0.004** | *P*=0.54 | *P*=0.46 | *P*=0.73 | *P*=0.50 | *P*=0.48 |
|  |  |  |  |  |  |  |  |
| Monounsaturated fat (gram) | r=0.06 | r=0.05 | r=−0.01 | r=−0.07 | r=−0.01 | r=0.04 | r=−0.00 |
|  | *P*=0.47 | *P*=0.57 | *P*=0.92 | *P*=0.40 | *P*=0.94 | *P*=0.67 | *P*=0.99 |
|  |  |  |  |  |  |  |  |
| Monounsaturated fat (E%) | r=0.01 | r=−0.21 | r=−0.04 | r=−0.02 | r=−0.07 | r=0.02 | r=0.14 |
|  | *P*=0.87 | ***P*=0.016** | *P*=0.66 | *P*=0.80 | *P*=0.51 | *P*=0.88 | *P*=0.17 |
|  |  |  |  |  |  |  |  |
| Polyunsaturated fat (gram) | r=0.08 | r=0.14 | r=−0.08 | r=−0.12 | r=−0.10 | r=−0.04 | r=0.10 |
|  | *P*=0.33 | *P*=0.12 | *P*=0.36 | *P*=0.16 | *P*=0.33 | *P*=0.73 | *P*=0.35 |
|  |  |  |  |  |  |  |  |
| Polyunsaturated fat (E%) | r=0.01 | r=0.07 | **r=−0.22** | **r=−0.19** | **r=−0.20** | r=−0.15 | **r=0.27** |
|  | *P*=0.89 | *P*=0.44 | ***P*=0.012** | ***P*=0.030** | ***P****=***0.042** | *P*=0.14 | ***P*=0.006** |
|  |  |  |  |  |  |  |  |
| Saturated fat (gram) | r=0.04 | r=0.01 | r=0.00 | r=−0.09 | r=0.04 | r=0.06 | r=−0.02 |
|  | *P*=0.62 | *P*=0.91 | *P*=0.98 | *P*=0.32 | *P*=0.66 | *P*=0.54 | *P*=0.83 |
|  |  |  |  |  |  |  |  |
| Saturated fat (E%) | r=−0.00 | r=−0.31 | r=0.03 | r=−0.02 | r=0.04 | r=0.07 | r=0.06 |
|  | *P*=0.98 | ***P*<.001** | *P*=0.69 | *P*=0.84 | *P*=0.68 | *P*=0.53 | *P*=0.55 |
| Pearson correlation coefficient (r) and p-value is presented.  **Abbreviations**: BMI, body mass index; E%, percent of total energy intake; SD, standard deviation; TIR, time in range (3.9–10 mmol/L). | | | | | | | |

**Supplemental Table S9.** Differences in dietary variables by sex (n=136).

|  | Female (n=66) | Male (n=70) | Mean difference (95% CI) | *P* |  |
| --- | --- | --- | --- | --- | --- |
| Total energy (kcal) | 1649 (680) | 1882 (814) | −234 (−489, 22) | 0.072 |  |
| Carbohydrates (g) | 166 (77) | 196 (102) | −28 (−59, 2) | 0.070 |  |
| Carbohydrates (E%) | 40.4 (7.0) | 41.2 (6.8) | −0.7 (−3.1, 1.6) | 0.53 |  |
| Total fat (g) | 68.5 (30.8) | 74.7 (32.7) | −6.2 (−17.0, 4.6) | 0.26 |  |
| Total fat (E%) | 37.3 (5.7) | 35.8 (5.8) | 1.5 (−0.5, 3.4) | 0.14 |  |
| Protein (g) | 73.3 (27.4) | 86.2 (35.1) | −12.9 (−23.6, −2.3) | 0.018 |  |
| Protein (E%) | 18.1 (2.7) | 18.5 (2.5) | −0.4 (−1.3, 0.5) | 0.36 |  |
| Fiber (g) | 23.3 (14.8) | 23.2 (15.3) | 0.0 (−5.1, 5.2) | 0.99 |  |
| Fiber (E%) | 2.7 (0.9) | 2.4 (0.8) | 0.3 (0.1, 0.6) | 0.020 |  |
| Wholegrain (g) | 60.6 (66.1) | 70.1 (68.9) | −9.5 (−32.4, 13.5) | 0.42 |  |
| Monounsaturated fatty acids (g) | 24.2 (11.1) | 26.4 (11.4) | −2.2 (−6.0, 1.7) | 0.27 |  |
| Monounsaturated fatty acids (E%) | 13.2 (2.3) | 12.7 (2.3) | 0.5 (−0.3, 1.3) | 0.22 |  |
| Polyunsaturated fatty acids (g) | 11.2 (7.2) | 10.4 (5.2) | 0.8 (−1.4, 2.9) | 0.47 |  |
| Polyunsaturated fatty acids (E%) | 5.8 (1.9) | 4.9 (1.6) | 0.9 (0.3, 115) | 0.003 |  |
| Saturated fatty acids (g) | 27.4 (11.5) | 31.4 (15.2) | −4.0 (−8.5, 0.6) | 0.086 |  |
| Saturated fatty acids (E%) | 15.1 (2.9) | 15.1 (3.3) | 0.1 (−1.0, 1.2) | 0.87 |  |
| Use of carbohydrate counting, % (n) * | 38.6% (n=32/83) | 36.0% (n=32/89) | 2.6 (−13.0, 18.2) | 0.75 |  |
| Descriptive data are presented as mean and standard deviation for numeric variables and as percentage, number, and total number of responders for categorical variables. Comparisons between groups were performed using independent samples t-test.  * Use of carbohydrate counting was retrieved from questionnaire data and complemented with information from the National Diabetes Register. Sample sizes were N = 83 and N = 89 for females and males, respectively.  **Abbreviations**: CI, confidence interval; E%, percent of total energy. | | | | | |

**Supplemental Table S10.** Differences in dietary and glucose variables by pump usage (n=135).

|  | Pump users (n=56) | MDI (n=79) | Mean difference (95% CI) | *P* |
| --- | --- | --- | --- | --- |
| Total energy (kcal) | 1666 (682) | 1838 (808) | −172 (−434, 90) | 0.20 |
| Carbohydrates (g) | 169 (80) | 191 (99) | −22 (−53, 10) | 0.18 |
| Carbohydrates (E%) | 40.5 (7.2) | 41.1 (6.7) | −0.6 (−3.0, 1.8) | 0.63 |
| Total fat (g) | 68.4 (31.4) | 73.8 (32.2) | −5.4 (−16.4, 5.6) | 0.33 |
| Total fat (E%) | 36.6 (5.8) | 36.4 (5.8) | 0.2 (−1.8, 2.2) | 0.83 |
| Protein (g) | 75.8 (29.0) | 82.4 (34.2) | −6.6 (−17.7, 4.5) | 0.24 |
| Protein (E%) | 18.5 (2.8) | 18.2 (2.4) | 0.3 (−0.6, 1.2) | 0.46 |
| Fiber (g) | 21.8 (11.3) | 24.1 (17.2) | −2.3 (−7.1, 2.5) | 0.35 |
| Fiber (E%) | 2.6 (0.9) | 2.5 (0.8) | 0.1 (−0.2, 0.4) | 0.42 |
| Wholegrain (g) | 54.0 (46.0) | 73.6 (78.9) | −19.5 (−40.9, 1.8) | 0.073 |
| Monounsaturated fatty acids (g) | 24.4 (11.4) | 26.0 (11.3) | −1.6 (−5.5, 2.3) | 0.42 |
| Monounsaturated fatty acids (E%) | 13.0 (2.4) | 12.8 (2.3) | 0.2 (−0.6, 1.0) | 0.59 |
| Polyunsaturated fatty acids (g) | 10.3 (5.2) | 11.0 (7.0) | −0.7 (−2.8, 1.3) | 0.48 |
| Polyunsaturated fatty acids (E%) | 5.5 (1.7) | 5.3 (1.8) | 0.2 (−0.4, 0.9) | 0.46 |
| Saturated fatty acids (g) | 28.0 (13.9) | 30.4 (13.4) | −2.3 (−7.1, 2.4) | 0.33 |
| Saturated fatty acids (E%) | 15.0 (3.0) | 15.1 (3.3) | −0.1 (−1.2, 1.0) | 0.85 |
| Use of carbohydrate counting, % (n) * | 53.4%  (39/73) | 24.5%  (24/98) | 28.9 (13.5, 44.4) | <.001 |
| Descriptive data are presented as mean and standard deviation for numeric variables and as percentage, number, and total number of responders for categorical variables. Comparisons between groups were performed using independent samples t-test.  * Use of carbohydrate counting was retrieved from questionnaire data and complemented with information from the National Diabetes Register. Sample sizes were N = 73 and N = 98 for pump users and MDI users, respectively.  **Abbreviations**: CI, confidence interval; E%, percent of total energy; MDI, multiple daily injections. | | | | |

**Supplemental Table S11.** Comparison of study participant characteristics with the Swedish National Diabetes Register (NDR) 2021.

|  |  |  | NDR annual data | | |
| --- | --- | --- | --- | --- | --- |
|  | Study population (n=191) | NDR annual report 2021  (n=48,719) | NU-hospital group  (Uddevalla and Trollhättan)  (n=1,380) | Uppsala University Hospital  (Akademiska Sjukhuset)  (n=1,780) | Vrinnevi Hospital,  Norrköping  (n=760) |
| HbA1c (mmol/mol) | 56.0 (12.5) | 59.3 | 56.8 | 58.1 | 59.4 |
| Age (years) | 48.3 (15.7) | 47.9 (17.9) | 49 | 47 | 47 |
| Female sex | 48.4% | 44.3% | 43.5% | 44.4% | 46% |
| BMI (kg/m^2^) | 26.6 (4.3) | 26.6 | 26.9 | 26.8 | 26.9 |
| Diabetes duration (years) | 28.3 (15.5) | 24.7 (15.9) | NA | NA | NA |
| SBP (mm Hg) | 126.4 (12.3) | 128.1 | 128.6 | 127.9 | 125.3 |
| DBP (mmHg) | 71.2 (9.7) | 74.9 | 70.8 | 74.7 | 74.5 |
| Pump users | 41% | 28% | 34% | 31% | 16% |
| **Abbreviations**: BMI, body mass index; DBP, diastolic blood pressure; NA, data not available; SBP, systolic blood pressure. | | | | | |

**Figure S1.** Flow chart of study participation, showing the number of individuals selected for participation, invited, excluded, and consenting to participate.

**
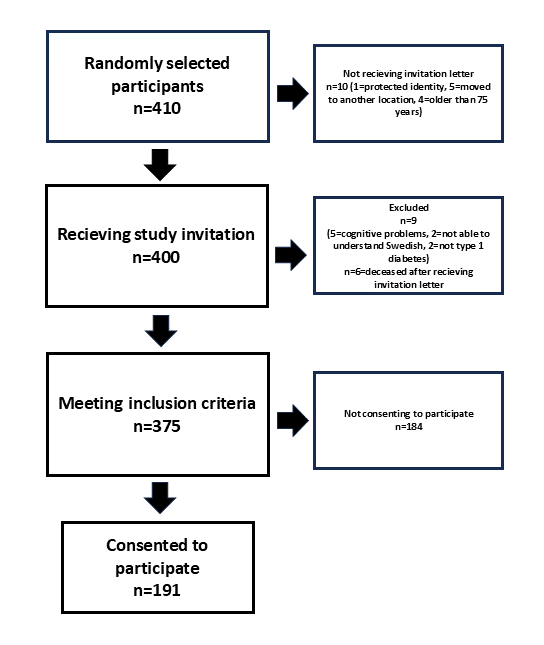
**
