# Supplementary material for: Diet and diet-related challenges with specific focus on carbohydrates and carbohydrate counting in adults with type 1 diabetes: a cross-sectional study
Source: BMJ Open. 2025 Nov 29;15(11):e101619. doi: 10.1136/bmjopen-2025-101619 (PMC12666139; doi:10.1136/bmjopen-2025-101619)
Supplement: online supplemental file 2 [file bmjopen-15-11-s002.docx]

# Questionnaire: Dietary Habits and Experiences of Diet-related Challenges in Type 1 Diabetes

## What type of diet do you usually follow?

Please select the option that best reflects how you usually eat, or choose 'Other diet' and briefly describe.

□ According to the Swedish National Food Agency’s recommendations for the general population (balanced diet according to the “plate model”)
□ Diabetes diet (whole grains, low glycaemic index (GI), vegetables, legumes, nuts, fish, lean meat, oils)
□ Mediterranean diet (lots of vegetables, olive oil, nuts, fish, lean meat)
□ Vegetarian diet (plant-based foods with addition of milk and/or eggs)
□ Vegan diet (only plant-based foods)
□ Low-carbohydrate diet (less carbohydrate, more fat and protein)
□ Very low-carbohydrate diet (almost no carbohydrate, high in fat and protein)
□ Other diet: __________

## 2. How important do you consider diet for maintaining good long-term blood glucose control?

□ Not at all important
□ Not very important
□ Fairly important
□ Very important
□ Extremely important
□ Don’t know/No opinion

## 3. Are you currently following a diet that you believe is the best for you? (for well-being and balanced glucose control)

□ Not at all true
□ Partly true
□ Neither true nor untrue
□ Largely true
□ Completely true
□ Don’t know/No opinion

## 4. Do you think you could achieve significantly better glucose control by changing how/what you eat?

□ Not at all true
□ Partly true
□ Neither true nor untrue
□ Largely true
□ Completely true
□ Don’t know/No opinion

## 5. Do you use carbohydrate counting?

Carbohydrate counting means estimating the number of grams of carbohydrate in a meal and adjusting the insulin dose accordingly.

□ I do not use carbohydrate counting / I don’t know what it is
□ I use carbohydrate counting without weighing food (estimate the amount)
□ I use carbohydrate counting by regularly weighing at least some types of food

□ I check nutritional labels to calculate carbohydrate amounts

□ I use apps/websites/books to calculate carbohydrate amounts

□ I use various mathematical rules such as the 100, 300, ror 500 rule to determine the correct insulin-to-carbohydrate ratio

□ I use the pumps carbohydrate counting functions to select the correct insulin dose

## 6. How important do you consider carbohydrate counting to be for achieving good long-term glucose control?

□ Not at all important
□ Not very important
□ Fairly important
□ Very important
□ Extremely important
□ Don’t know/No opinion

## 7. How easy/difficult do you find carbohydrate counting? (to calculate the correct insulin dose for meals)

□ Very easy
□ Easy
□ Fairly easy
□ Neither easy nor difficult
□ Quite difficult
□ Difficult
□ Very difficult
□ Don’t know/No opinion

## 8. Have you received information/education on carbohydrate counting from healthcare (nurse, physician, dietitian) during the past year?

□ Yes
□ No
□ Don’t know

## 9. Do you consciously try to reduce the amount of carbohydrates at meals to achieve better glucose control? (examples of carbohydrates: rice, pasta, bread, grains, root vegetables, potatoes, fruit, sugar etc.)

□ Not at all true
□ Partly true
□ Neither true nor untrue
□ Largely true
□ Completely true
□ Don’t know/No opinion

## 10. Do you usually choose whole grain products for bread/pasta/flour? (e.g., whole grain bread, graham flour, whole grain pasta)

□ Not at all true
□ Partly true
□ Neither true nor untrue
□ Largely true
□ Completely true
□ Don’t know/No opinion

## 11. Is there any food/meal that you avoid or eat less often because it does not work with your diabetes (leads to poor glucose control) but that you wish you could eat more of?

□ Yes
□ No
□ Don’t know/No opinion

If yes, which? __________

## 12. How easy/difficult do you find it to calculate the correct insulin dose for a meal and stay within your target glucose range 1.5–2 hours afterwards, also avoiding later highs/lows, when eating the following carbohydrate-rich foods?

Scale: Very easy – Easy – Fairly easy – Neither easy nor difficult – Fairly difficult – Difficult – Very difficult – Don’t know/No opinion

□ Pasta
□ Whole grain pasta
□ Rice
□ Brown rice/whole grain rice
□ Cooked grains (wheat berries, barley, bulgur)
□ White bread (e.g., baguette, sandwich loaf)
□ Semi-whole grain bread (e.g., lingongrova)
□ Whole grain bread (e.g., Danish rye bread)
□ Sourdough bread
□ Pizza
□ Boiled potatoes
□ Fried potatoes/French fries
□ Mashed potatoes
□ Other: __________

## 13. Have you received sufficient information/help with diet from healthcare (e.g., diabetes nurse, physician, dietitian) since your diabetes diagnosis?

□ Not at all true
□ Partly true
□ Neither true nor untrue
□ Largely true
□ Completely true
□ Don’t know/No opinion

## 14. When was the last time you received dietary information/help from a dietitian?

□ 1–3 months ago
□ 3–6 months ago
□ 6–12 months ago
□ More than 12 months ago
□ More than 2 years ago
□ More than 5 years ago
□ Have never met a dietitian
□ Don’t know

## 15. Do you currently wish to receive information/help with diet from a dietitian?

□ Not at all true
□ Partly true
□ Neither true nor untrue
□ Largely true
□ Completely true
□ Don’t know/No opinion

## 16. How easy or difficult do you find it to calculate the correct insulin dose in the following situations? (to achieve good glucose control about 1.5–2 hours afterwards and beyond)

Scale: Very easy – Easy – Fairly easy – Neither easy nor difficult – Fairly difficult – Difficult – Very difficult – Don’t know/No opinion

□ At breakfast
□ At lunch
□ At dinner
□ At snack
□ After hypoglycaemia
□ When blood glucose is high before the meal
□ At parties (prolonged eating, alcohol)
□ At restaurants (don’t know what’s in the food)
□ In connection with exercise
□ Other occasion (please specify): __________

## 17. To avoid low glucose after meals, how important do you consider the following factors?

Scale: Not at all important – Not very important – Fairly important – Very important – Extremely important – Don’t know/No opinion

□ Amount of carbohydrate in the meal (e.g., bread, rice, pasta, potatoes, sugar)
□ Type of carbohydrate (e.g., fast vs. slow)
□ If the meal contains a lot of fat
□ If the meal contains a lot of protein (e.g., meat, fish, eggs)
□ If the meal contains a lot of fibre (e.g., whole grains, flaxseeds)
□ Amount of insulin you dose for the meal
□ Type of bolus insulin for the meal (e.g., split dose or extended dose)
□ Timing of insulin dose for the meal
□ Physical activity/exercise before the meal
□ If you will exercise after the meal
□ Whether you checked glucose levels before the meal
□ How you feel at the time of the meal (stress, infection, etc.)
□ Other: __________

## 18. To avoid high blood glucose after meals, how important do you consider the following factors?

Scale: Not at all important – Not very important – Quite important – Very important – Extremely important – Don’t know/No opinion

□ Amount of carbohydrate in the meal
□ Type of carbohydrate
□ If the meal contains a lot of fat
□ If the meal contains a lot of protein
□ If the meal contains a lot of fibre
□ Amount of insulin you dose for the meal
□ Type of bolus insulin for the meal
□ Timing of insulin dose for the meal
□ Physical activity/exercise before the meal
□ If you will exercise after the meal
□ Whether you checked glucose levels before the meal
□ How you feel at the time of the meal (stress, infection, etc.)
□ Other: __________

## 19. What is your highest level of education?

Please select the option that best corresponds to your highest completed level of education (only one answer).

□ Did not complete compulsory school or equivalent basic education
□ Compulsory school, elementary school or equivalent (up to 9 years)
□ Secondary school, upper secondary, folk high school (general adult education college), vocational training or equivalent
□ University or college degree
