# Supplementary material for: Diet and diet-related challenges with specific focus on carbohydrates and carbohydrate counting in adults with type 1 diabetes: a cross-sectional study
Source: BMJ Open. 2025 Nov 29;15(11):e101619. doi: 10.1136/bmjopen-2025-101619 (PMC12666139; doi:10.1136/bmjopen-2025-101619)
Supplement: Supplementary file 3 [file bmjopen-2025-101619.reviewer_comments.pdf]

PEER REVIEW HISTORY

BMJ Open publishes all reviews undertaken for accepted manuscripts. Reviewers are asked to complete a checklist review form and are provided with free text boxes to elaborate on their assessment. These free text comments are reproduced below.

ARTICLE DETAILS

Title (Provisional)

Diet and diet-related challenges with specific focus on carbohydrates and carbohydrate counting in adults with type 1 diabetes: a cross-sectional study

Authors

Sterner Isaksson, Sofia; Hellman, Jarl; Wijkman, Magnus; Imberg, Henrik; Ólafsdóttir, Arndís Finna; Axelsen, Mette; Lind, Marcus

---

VERSION 1 - REVIEW

Reviewer 1

Name Bengtsen, MB

Affiliation Aarhus University Hospital, Endocrinology and Internal Medicine

Date 13-May-2025

COI None

Deal all,

I have some specific comments aimed at clarifying some issues in the manuscript.

Isaksson et at investigated dietary habits, diabetes-related challenges, and the need for dietary support among Swedish adults with type 1 diabetes (T1D) using a survey of 191 participants. Results showed that many participants struggled with carbohydrate counting and often relied on visual estimation. Their diets typically included lower carbohydrate and fiber intake, but higher saturated fat intake, deviating from recommended guidelines. About 48% had reduced carbohydrate consumption, and 30% avoided certain carbohydrate-rich foods due to glucose management difficulties. Over 40% felt they lacked adequate dietary guidance from healthcare providers, and many expressed a desire for dietitian support. They conclude that the findings underscore the importance of personalized dietary counseling in T1D care.

Overall comments

Strengths of the study include randomly choosing people with T1D to participate and involving patients in the proces of designing questionnaries (page 7 line 10).

Specfic comments

Page 4 Line 16: ”Diet plays a central role in glucose management, yet many individuals with T1D face

challenges related to insulin dosing for specific meals." I suggest adding a reference backing this statement up.

Page 4 Line 28-36. "Current dietary guidelines suggest that a range of macronutrient distributions can be effective for glucose control (6). Observational studies (7-9) and small clinical trials (10-13) have found associations between reduced carbohydrate intake and improved glucose management, while also highlighting negative effects on diet quality and lipids when fat intake increases (14, 15). Recent evidence supports a moderate carbohydrate intake (30 percent of energy) as beneficial for glucose control (16)". It would strengthen the introduction if you state whether all the references are conducted in studies in people with type 1 diabetes.

Page 6 line 26. "Inclusion criteria included adults aged 18–75 years with type 1 diabetes and diabetes duration of more than one year"

Please elaborate why you choose to exclude people above 75 years if age and <1 year of DM duration ?

Page 8 line 31. The accurate and correct time in range internationally is the range of 3,9 mmol/L – 10 mmol/L (https://diabetesjournals.org/clinical/article/38/5/439/32320/Time-in-Range-How-to-Measure-It-How-to-Report-It)

Page 12 line 49. "Mean carbohydrate intake in this population (41 E%) was lower than the generally recommended 45–60 E% (6, 25, 26) but consistent with findings from other diabetes

populations in Sweden (16, 27) and Australia (28) of 41-43 E%,". Please clarify what the E stands for.

**Reviewer**	**2**
**Name**	**Barouti, Afroditi**
**Affiliation**	**Karolinska Hospital**
**Date**	**22-Sep-2025**
**COI**	**None**

Please see the attached file with detailed review and comments for all sections. Overall, I highly support the publication of this manuscript. Please do not be alarmed by the number of comments, most are minor with only few major revision required, mainly in the Discussion and Conclusion.

Review of manuscript entitled "**Diet and diet related challenges with specific focus on carbohydrates and carbohydrate counting in adults with type 1 diabetes: insights from a Swedish survey**"

The study by Isaksson et al was a cross-sectional study investigating dietary intake and diet-related challenges with regard to glycemic control in a sample of 191 adults with type 1 diabetes in Sweden. They used a validated FFQ and a newly developed questionnaire to assess diet-

related strategies and challenges. They also explored associations between participant characteristics and nutritional intake.

Their results showed that study participants had an average intake of 41% of energy from carbohydrates (183 g/d), 23 g fiber/day and 15% of energy SFAs. They found carbohydrate counting mostly difficult, using mostly visual assessment of carbs, almost half reported reducing total carb intakes and 60% avoided specific carbohydrate-rich foods (high in simple sugars or processed carbs and/or fats) (Fig 2). The majority found diet very important for maintaining long-term glucose control but only 40% reported receiving partially or fully sufficient information/help from healthcare related to their diet. Interestingly, only 35% wished to receive information/help with their diet from a dietitian.

This is a highly valuable study that sheds light on dietary intake, strategies, and challenges in people with type 1 diabetes, an area that is still underexplored. The work makes an important contribution, and I fully support its publication as the findings provide meaningful insights for both research and clinical practice.

Most of my suggestions are minor, aimed at strengthening the manuscript, though a few are more substantial. The dietary assessment methods are generally appropriate, but the questionnaire on diet-related challenges lacks additional useful information. The main issue is in the discussion, where intake is compared to general population guidelines rather than diabetes-specific recommendations and some other interpretation issues. Finally, the conclusions should more closely reflect the results.

Revisions per section:

Abstract

1) The aim could be clearer to the reader. I suggest specifying that the study investigates dietary intake and diet-related challenges regarding glucose management, rather than just “diet,” to align with the terminology used in the main text.
2) Lines 41 (“inconsistent with dietary recommendations”) and 55 (“lower intake of carbohydrates”) should be rephrased. As discussed later, there are no specific macronutrient targets, particularly %EI from carbohydrates, for people with diabetes. Fiber and saturated fat intake are indeed inconsistent with recommendations.
3) Line 43 states that “30% avoided certain carbohydrate-rich foods due to glucose management difficulties.” Based on Figure 2A, this appears closer to 60%, please check and clarify.
4) Page 3, line 3, conclusion: “many expressed a significant interest in dietitian support.” Reporting 30–35% as “many” may be misleading, as this is a smaller proportion compared to participants not wishing dietitian contact or being neutral.

Introduction

1) Page 4, third paragraph, first line: Please clarify that you are referring to current nutritional guidelines for people with diabetes. You could also include the ADA guidelines. Please also refer to the most recent meta-analysis of RCTs on dietary patterns in people with T1D by Zeng et al in eClinicalMedicine https://www.sciencedirect.com/science/article/pii/S2589537025001543, which highlights that both quantity and quality of carbohydrates are important for glucose management in T1D; particularly increasing fiber intake while reducing other carbohydrate sources seems to show the most benefits for glycemic control and variability in T1D.
2) Lines 33–34: The statement "while also highlighting negative effects on diet quality and lipids when fat intake increases (14, 15)" is potentially misleading. The study by Levran et al demonstrated lower fiber intake and the need for nutritional monitoring to maintain fiber while reducing other carbohydrates. The study by Leow et al was observational and focused on ketogenic diets; therefore, conclusions about negative effects on lipids from low-carbohydrate diets cannot be drawn. A more accurate framing would emphasize the potential risk of insufficient fiber intake and high saturated fat intake associated with low-carbohydrate diets, which is consistent with the findings of the current study.

Design and Methods

1) Page 7, lines 3–5: Please clarify the recall period for the FFQ (e.g., 3, 6, or 12 months), as this is important for accounting for seasonal variability. Additionally, indicate briefly whether standard portion sizes were used for nutrient calculations and specify which food composition databases were applied, for clarity and reproducibility.
2) Page 7, lines 49–50: Rather than stating "all adults with T1D," please include the most recent coverage percentage for the Swedish National Diabetes Register (NDR) to provide accurate context.

Results

1) Page 9, line 27: It appears that 35 participants were excluded from the initial 410, resulting in 375 people receiving the questionnaires. Please review the numbers provided in the text and in Figure S1, as the exclusions do not seem to match.
2) Do the authors have access to participant characteristics for all 375 individuals who were sent questionnaires? If so, it would be informative to compare characteristics between those who responded (n=191, 51%) and non-responders (49%), given the relatively high non-response rate. This could strengthen the interpretation and discussion of the findings.
3) Page 10, line 27: The statement "over 30%" is vague. Please provide a specific number or report it as "approximately 30%" to avoid giving the impression that it is substantially higher than other categories.
4) Page 11, lines 47 and 54: Education data are reported, but the source is unclear. Please specify in the Methods the source of these data.
5) It would be valuable to provide results on dietary intake by food group based on FFQ data. For example, servings or grams of fruits and vegetables, whole grains, dairy, legumes, nuts, and seeds etc. These data would help assess diet quality in the context of diabetes nutrition recommendations, as such foods are emphasized. It is ok if it not possible, but it would increase the quality of the paper.
6) The correlation section of the Results could be shortened to better focus on the main findings of the study.

Discussion and conclusions

1) First paragraph line 28-31, the use and importance of carbohydrate counting. I suggest revisiting how the question on carbohydrate counting was phrased. In the results you report that "Among those who used it, 53% relied on visual estimation… while the remaining participants used more advanced methods." This implies that even those relying only on visual estimation were categorized as carb-counting users. If so, it is not surprising that most

participants also considered carb counting important for glycemic control. Please double-check the question wording and clarify this in both the results and Table S6.

2) Lines 31 "Mean carbohydrate intake was relatively low (41% of total energy)", and 38 "However, only 8% reported following a low-carbohydrate diet.": Since no definition of "low carbohydrate" was provided in the questionnaire or the manuscript, it is difficult to compare "41% of energy from carbohydrates" and the self-reported 8% following a low-carb diet. To avoid misinterpretation, I recommend rephrasing to something like: Mean carbohydrate intake was 41% of total energy, with many participants intentionally restricting carbohydrates and avoiding foods like sushi, pizza, sweets, and sweet beverages. However, only 8% reported following a low-carbohydrate diet, highlighting the well-known challenges in defining and classifying carbohydrate restricted diets.
3) **Major revision Discussion paragraph 2**: Please compare participants' intake to the dietary recommendations for people with diabetes rather than the general population. This would change part of the interpretations of the results as people with diabetes and particularly T1D are not required to follow the "45-60% of energy from carbs" recommendation. Instead, many different patterns of different macronutrient composition can be recommended if fiber intake is adequate and saturated fat remains low. Both DNSG and ADA guidelines emphasize flexibility here, so please revise this paragraph accordingly. A low carb diet can be high in fibers and low in SFA focusing on unsaturated fats if properly designed, see the meta-analysis of Zeng et al which also strengthens the perceived better glycemic control with lower total carb intakes and fiber-rich foods observed in your study. You could also expand on whether participants consumed the recommended food groups (e.g., whole grains, legumes, nuts, vegetables) or tended toward processed/simple carbs. Finally, you could possibly mention that hypoglycemia management often necessitates intake of simple sugars (and sometimes foods rich in SFAs like sweets, desserts, chocolate), which complicates dietary quality assessments in T1D populations. Please also revise page 13 lines 44-47 (you can refer to diabetes guidelines for the SFA cutoff of 10%).
4) Page 14 lines 54-60 and page 15 lines 1-10 the paragraph on implications. My conclusion from your results is somewhat different. As I interpret them, most participants felt that diet-related information from healthcare was insufficient, yet only ~35% wished to meet with a dietitian, despite recognizing the importance of diet. This suggests a gap between patients' needs and healthcare, particularly dietetic support. It might have been valuable to include open questions on barriers to dietitian visits. If relevant, you could check whether literature exists on barriers to accessing dietitians among people with T1D.
5) **Limitations**: The new questionnaire did not collect dietary data specifically related to hypoglycemia management (snacks, foods, beverages, and their frequency), which is crucial in this population. It did not provide clear definitions of dietary patterns (e.g., Swedish Food Agency's guidelines, Mediterranean, low carb), leaving interpretation to participants. Some questions (e.g., answering "partially" about wanting to meet a dietitian) could be ambiguous.

   A pilot test for content validity and comprehension would have been valuable. Please consider adding these as limitations.
6) Conclusions page 16 lines 25-27 and 36-39: please revise according to the points made above (not having received sufficient dietary information is not the same as wishing for help or guidance from healthcare)

Figures and Tables

1) Table S5: The question is how often you usually eat the following meals and under breakfast, lunch and dinner there is the option of "several times per day". Is this how it was asked? Did you mean "several times per week" maybe?
2) Table S6: please revise the format to ensure clarity (see first point in Discussion). Are there missing answers as well, should the % add up to 100? A bit unclear.

VERSION 1 - AUTHOR RESPONSE

Reviewer: 1
Dr. MB Bengtsen, Aarhus University Hospital
Comments to the Author:

Overall comments
Strengths of the study include randomly choosing people with T1D to participate and involving patients in the proces of designing questionnaries (page 7 line 10).

Specific comments

Page 4 Line 16: ”Diet plays a central role in glucose management, yet many individuals with T1D face challenges related to insulin dosing for specific meals.” I suggest adding a reference backing this statement up.

Response: Thank you for this comment. We have now added references to back up the statement (references 6 and 7).

Page 4 Line 28-36. ”Current dietary guidelines suggest that a range of macronutrient distributions can be effective for glucose control (6). Observational studies (7-9) and small clinical trials (10-13) have found associations between reduced carbohydrate intake and improved glucose management, while also highlighting negative effects on diet quality and lipids when fat intake increases (14, 15). Recent evidence supports a moderate carbohydrate intake (30 percent of energy) as beneficial for glucose control (16)”. It would strengthen the introduction if you state whether all the references are conducted in studies in people with type 1 diabetes.

Response: Thank you for this important comment. We have now re-written this part a bit due to comments from reviewer 2 and also making it more clear that all studies refer to type 1 diabetes.

Page 6 line 26. ”Inclusion criteria included adults aged 18–75 years with type 1 diabetes and diabetes duration of more than one year”
Please elaborate why you choose to exclude people above 75 years if age and
<1 year of DM duration ?>

Response: Thank you for this comment. We chose to include only participants with more than one year of diabetes duration to ensure that they passed the initial treatment adjustment phase and reached a more stable stage of their diabetes management. The upper age limit of 75 years was set because all study questionnaires were distributed digitally, and we anticipated that individuals in the highest age range might experience difficulties with the required technology, which could have affected data quality and response rates. We have clarified this in the revised manuscript in the methods section.

Page 8 line 31. The accurate and correct time in range internationally is the range of 3,9 mmol/L – 10 mmol/L (https://diabetesjournals.org/clinical/article/38/5/439/32320/Time-in-Range-How-to-Measure-It-How-to-Report-It)

Response: Thank you for noticing this typing error. We have now corrected it to be 3.9-10 mmol/L.

Page 12 line 49. ”Mean carbohydrate intake in this population (41 E%) was lower than the generally recommended 45–60 E% (6, 25, 26) but consistent with findings from other diabetes populations in Sweden (16, 27) and Australia (28) of 41-43 E%,”. Please clarify what the E stands for.

Response: The E stands for energy and macronutrients are presented in percent of energy (E%). This is now correctly explained in the section “Data collection”.

Reviewer: 2
Dr. Afroditi Barouti, Karolinska Hospital
Comments to the Author:
Please see the attached file with detailed review and comments for all sections. Overall, I highly support the publication of this manuscript. Please do not be alarmed by the number of comments, most are minor with only few major revision required, mainly in the Discussion and Conclusion.

Review of manuscript entitled “**Diet and diet related challenges with specific focus on carbohydrates and carbohydrate counting in adults with type 1 diabetes: insights from a Swedish survey**”

The study by Isaksson et al was a cross-sectional study investigating dietary intake and diet-related challenges with regard to glycemic control in a sample of 191 adults with type 1 diabetes in Sweden. They used a validated FFQ and a newly developed questionnaire to assess diet-related strategies and challenges. They also explored associations between participant characteristics and nutritional intake.

Their results showed that study participants had an average intake of 41% of energy from carbohydrates (183 g/d), 23 g fiber/day and 15% of energy SFAs. They found carbohydrate counting mostly difficult, using mostly visual assessment of carbs, almost half reported reducing total carb intakes and 60% avoided specific carbohydrate-rich foods (high in simple sugars or processed carbs and/or fats) (Fig 2). The majority found diet very important for maintaining long-term glucose control but only 40% reported receiving partially or fully sufficient information/help from healthcare related to their diet. Interestingly, only 35% wished to receive information/help with their diet from a dietitian.

This is a highly valuable study that sheds light on dietary intake, strategies, and challenges in people with type 1 diabetes, an area that is still underexplored. The work makes an important contribution, and I fully support its publication as the findings provide meaningful insights for both research and clinical practice.

Most of my suggestions are minor, aimed at strengthening the manuscript, though a few are more substantial. The dietary assessment methods are generally appropriate, but the questionnaire on diet-related challenges lacks additional useful information. The main issue is in the discussion, where intake is compared to general population guidelines rather than

diabetes-specific recommendations and some other interpretation issues. Finally, the conclusions should more closely reflect the results.

Revisions per section:

Abstract

1) The aim could be clearer to the reader. I suggest specifying that the study investigates dietary intake and diet-related challenges regarding glucose management, rather than just "diet," to align with the terminology used in the main text.

Response: Thank you for this wise suggestion. We have now changed the aim according to your suggestion.

2) Lines 41 ("inconsistent with dietary recommendations") and 55 ("lower intake of carbohydrates") should be rephrased. As discussed later, there are no specific macronutrient targets, particularly %EI from carbohydrates, for people with diabetes. Fiber and saturated fat intake are indeed inconsistent with recommendations.

Response: We agree and have removed carbohydrates from that section to make it clear that it is fiber and saturated fats that are lower than recommendations.

3) Line 43 states that "30% avoided certain carbohydrate-rich foods due to glucose management difficulties." Based on Figure 2A, this appears closer to 60%, please check and clarify.

Response: Thank you for noticing this error. We have now changed the number to the correct 61%.

4) Page 3, line 3, conclusion: "many expressed a significant interest in dietitian support." Reporting 30–35% as "many" may be misleading, as this is a smaller proportion compared to participants not wishing dietitian contact or being neutral.

Response: Thank you for this comment. We agree that the wording may be misleading, although it is still a relatively large number of participants if relating it to the patient population in the clinic. We have reformulated the wording to "one-third", so it is no longer misleading.

Introduction

1) Page 4, third paragraph, first line: Please clarify that you are referring to current nutritional guidelines for people with diabetes. You could also include the ADA guidelines. Please also refer to the most recent meta-analysis of RCTs on dietary patterns in people with T1D by Zeng et al in eClinicalMedicine https://www.sciencedirect.com/science/article/pii/S2589537025001543, which highlights that both quantity and quality of carbohydrates are important for glucose management in T1D; particularly increasing fiber intake while reducing other carbohydrate sources seems to show the most benefits for glycemic control and variability in T1D.

Response: Thank you for pointing this out. We have now added that the recommendations are for adults with diabetes and included the ADA recommendations as reference and rewritten the whole section to be clearer.

2) Lines 33–34: The statement "while also highlighting negative effects on diet quality and lipids when fat intake increases (14, 15)" is potentially misleading. The study by Levran et al demonstrated lower fiber intake and the need for nutritional monitoring to maintain fiber while reducing other carbohydrates. The study by Leow et al was observational and focused on ketogenic diets; therefore, conclusions about negative effects on lipids from low-carbohydrate diets cannot be drawn. A more accurate framing would emphasize the potential risk of insufficient fiber intake and high saturated fat intake associated with low-carbohydrate diets, which is consistent with the findings of the current study.

Response: Thank you for pointing out that this section was a bit unclear. We have now changed the text to be more clear about what we mean, that carbohydrates affect glucose levels and fat quality may affect lipid profiles.

Design and Methods

1) Page 7, lines 3–5: Please clarify the recall period for the FFQ (e.g., 3, 6, or 12 months), as this is important for accounting for seasonal variability. Additionally, indicate briefly whether standard portion sizes were used for nutrient calculations and specify which food composition databases were applied, for clarity and reproducibility.

Response: The Meal-Q FFQ is designed to reflect "the past few months" according to the description, but it does not explicitly detail the exact number of months back in time. "The past few" is most likely reflecting the past 2-3 months and we have added <3 months for clarity in the manuscript. We have also added information about portion sizes and nutrient database in the section about Meal-Q in the methods section.

2) Page 7, lines 49–50: Rather than stating "all adults with T1D," please include the most recent coverage percentage for the Swedish National Diabetes Register (NDR) to provide accurate context.

Response: Thank you for this suggestion. We have now added the approximate number (49 000) of adults included in NDR 2021.

Results

1) Page 9, line 27: It appears that 35 participants were excluded from the initial 410, resulting in 375 people receiving the questionnaires. Please review the numbers provided in the text and in Figure S1, as the exclusions do not seem to match.

Response: Thank you for noticing this. The figure is correct, but the number 25 was wrong and should have been 35. We have now corrected the numbers and changed the text in that section to make it clearer.

2) Do the authors have access to participant characteristics for all 375 individuals who were sent questionnaires? If so, it would be informative to compare characteristics between those who responded (n=191, 51%) and non-responders (49%), given the relatively high non-response rate. This could strengthen the interpretation and discussion of the findings.

Response: Thank you for this valuable comment. Unfortunately, we do not have access to that data. That is why we instead made comparisons of our responders with some of the main characteristics of the NDR 2021 population.

3) Page 10, line 27: The statement “over 30%” is vague. Please provide a specific number or report it as “approximately 30%” to avoid giving the impression that it is substantially higher than other categories.

Response: Thank you for pointing this out. We have now changed the percentage units to be specific here, and in the manuscript overall.

4) Page 11, lines 47 and 54: Education data are reported, but the source is unclear. Please specify in the Methods the source of these data.

Response: Thank you for this comment. We asked about educational level in the researcher-developed questionnaire. This is now described in the methods section, and the questionnaire is also provided as supplement.

5) It would be valuable to provide results on dietary intake by food group based on FFQ data. For example, servings or grams of fruits and vegetables, whole grains, dairy, legumes, nuts, and seeds etc. These data would help assess diet quality in the context of diabetes nutrition recommendations, as such foods are emphasized. It is ok if it not possible, but it would increase the quality of the paper.

Response: Thank you for this suggestion. We agree that it would be of interest to group all the questions regarding these food groups and look at how many standard portions per day or week the participants consumed. This might be a theme for a future manuscript.

6) The correlation section of the Results could be shortened to better focus on the main findings of the study.

Response: Thank you for this comment. We have now shortened this section.

Discussion and conclusions

1) First paragraph line 28-31, the use and importance of carbohydrate counting. I suggest revisiting how the question on carbohydrate counting was phrased. In the results you report that “Among those who used it, 53% relied on visual estimation… while the remaining participants used more advanced methods.” This implies that even those relying only on visual estimation were categorized as carb-counting users. If so, it is not surprising that most participants also considered carb counting important for glycemic control. Please double-check the question wording and clarify this in both the results and Table S6.

Response: Thank you for this comment. The question was asked to investigate whether carbohydrate counting was used, and if used in which form (advanced or not). We have now changed the wording to that part in the paragraph as well as in the results section trying to make it clear that it was carbohydrate counting overall, and not specifically advanced carbohydrate counting that was considered important for glycemic control by participants, and that the question was answered by all participants.

2) Lines 31 “Mean carbohydrate intake was relatively low (41% of total energy)”, and 38 “However, only 8% reported following a low-carbohydrate diet.”: Since no definition of “low

carbohydrate" was provided in the questionnaire or the manuscript, it is difficult to compare "41% of energy from carbohydrates" and the self-reported 8% following a low-carb diet. To avoid misinterpretation, I recommend rephrasing to something like: Mean carbohydrate intake was 41% of total energy, with many participants intentionally restricting carbohydrates and avoiding foods like sushi, pizza, sweets, and sweet beverages. However, only 8% reported following a low-carbohydrate diet, highlighting the well-known challenges in defining and classifying carbohydrate restricted diets.

Response: Thank you for this comment. We have now rephrased this paragraph to make it clearer and more concise.

3) **Major revision Discussion paragraph 2**: Please compare participants' intake to the dietary recommendations for people with diabetes rather than the general population. This would change part of the interpretations of the results as people with diabetes and particularly T1D are not required to follow the "45-60% of energy from carbs" recommendation. Instead, many different patterns of different macronutrient composition can be recommended if fiber intake is adequate and saturated fat remains low. Both DNSG and ADA guidelines emphasize flexibility here, so please revise this paragraph accordingly. A low carb diet can be high in fibers and low in SFA focusing on unsaturated fats if properly designed, see the meta-analysis of Zeng et al which also strengthens the perceived better glycemic control with lower total carb intakes and fiber-rich foods observed in your study. You could also expand on whether participants consumed the recommended food groups (e.g., whole grains, legumes, nuts, vegetables) or tended toward processed/simple carbs. Finally, you could possibly mention that hypoglycemia management often necessitates intake of simple sugars (and sometimes foods rich in SFAs like sweets, desserts, chocolate), which complicates dietary quality assessments in T1D populations. Please also revise page 13 lines 44-47 (you can refer to diabetes guidelines for the SFA cutoff of 10%).

Response: Thank you for this important comment. We have now changed this paragraph to only compare with diabetes populations and recommendations. We also added fiber intake in g/MJ and sucrose in E% in the results table to be able to compare better with guidelines and added a sentence about hypoglycemia management, as suggested.

4) Page 14 lines 54-60 and page 15 lines 1-10 the paragraph on implications. My conclusion from your results is somewhat different. As I interpret them, most participants felt that diet-related information from healthcare was insufficient, yet only ~35% wished to meet with a dietitian, despite recognizing the importance of diet. This suggests a gap between patients' needs and healthcare, particularly dietetic support. It might have been valuable to include open questions on barriers to dietitian visits. If relevant, you could check whether literature exists on barriers to accessing dietitians among people with T1D.

Response: Thank you for this valuable comment. We agree that it might have been valuable to further explore how the participants really felt regarding contacts with dietitians. However, we interpreted the results in the way that many participants (40%) felt that they haven't received sufficient guidance on diet from healthcare professionals since their diagnosis, and that 30% were interested in more help from a dietitian right now. That is a relatively high number, indicating a need to strengthen access to dietitians in routine care, we believe. However, we have now changed the wording in conclusion to be more balanced regarding the numbers. We have also added a section describing possible barriers, including references.

5) **Limitations**: The new questionnaire did not collect dietary data specifically related to hypoglycemia management (snacks, foods, beverages, and their frequency), which is crucial in this population. It did not provide clear definitions of dietary patterns (e.g., Swedish Food Agency's guidelines, Mediterranean, low carb), leaving interpretation to participants. Some questions (e.g., answering "partially" about wanting to meet a dietitian) could be ambiguous.

A pilot test for content validity and comprehension would have been valuable. Please consider adding these as limitations.

Response: Thank you for this comment. A new questionnaire can of course have its limits, although we gave the questionnaire to a number of people working with diabetes (including several dietitians) for feedback before we started the study. We have added this as a limitation in the proper section under strengths and limitations.

6) Conclusions page 16 lines 25-27 and 36-39: please revise according to the points made above (not having received sufficient dietary information is not the same as wishing for help or guidance from healthcare)

Response: Thank you for pointing out that this section was not clear. We have now changed the wording in this section, separating the different results to make it clearer.

Figures and Tables

1) Table S5: The question is how often you usually eat the following meals and under breakfast, lunch and dinner there is the option of "several times per day". Is this how it was asked? Did you mean "several times per week" maybe?

Response: Thank you for noticing this error, we have now changed it to the correct "several times per week".

2) Table S6: please revise the format to ensure clarity (see first point in Discussion). Are there missing answers as well, should the % add up to 100? A bit unclear.

Response: Thank you for noticing this error. We have now corrected the table and the results in the manuscript with the correct numbers. We have also improved the layout of Table S6 and highlighted the specific question to make the separation between questions clearer.

VERSION 2 - REVIEW

**Reviewer**	**1**
**Name**	**Bengtsen, MB**
**Affiliation**	**Aarhus University Hospital, Endocrinology and Internal Medicine**
**Date**	**29-Oct-2025**
**COI**	

I am pleased with the changes made by the authors

**Reviewer**	**2**
**Name**	**Barouti, Afroditi**
**Affiliation**	**Karolinska Hospital**
**Date**	**08-Nov-2025**
**COI**	

A small comment on line 342, you can remove carbohydrates from the following sentence according to your previous revisions: Participants' dietary intake did not align to dietary guidelines regarding carbohydrates, fibre and saturated fat intake, further underscoring the potential value of dietitian-led support.
